# KLF5 activates lncRNA DANCR and inhibits cancer cell autophagy accelerating gastric cancer progression

**DOI:** 10.1038/s41525-021-00207-7

**Published:** 2021-09-21

**Authors:** Zhiyi Cheng, Guiyuan Liu, Chuanjiang Huang, Xiaojun Zhao

**Affiliations:** Department of Gastrointestinal surgery, Hospital Afiliated 5 to Nantong University (Taizhou People’s Hospital), Taizhou, 225300 PR China

**Keywords:** Gastric cancer, Gastric cancer

## Abstract

Cancer cell autophagy has been associated with the progression of gastric cancer (GC), but involvement of long noncoding RNAs (lncRNAs) remains unclear. Initial bioinformatics analysis has identified abnormally highly expressed KLF5 in GC, as well as the predicted regulatory mechanism associating with lncRNA DANCR, miR-194, and AKT2. The expression of KLF5, DANCR, and AKT2 in GC tissue was upregulated, and the expression of miR-194 was downregulated. We knocked KLF5 down and manipulated the expression of DANCR, miR-194, and AKT2 to characterize their roles in GC cell viability, autophagy, and apoptosis. The mechanistic investigations revealed that KLF5 activated the transcription of DANCR in the promoter region and elevated its expression. DANCR acted as a miR-194 sponge to repress its expression in GC. MiR-194 targeted and inhibited AKT2 expression. Silencing KLF5 augmented GC cell autophagy, apoptosis and impeded its viability through the DANCR/miR-194/AKT2 axis. The tumor-inhibiting properties of KLF5 knockdown were substantiated in vivo. Together, our study uncovered the oncogenic role of KLF5-dependent lncRNA DANCR transcription in GC in vivo and in vitro, which implicates the miR-194/AKT2 axis in tumor growth regulation, and it may be a potential therapeutic target for human GC.

## Introduction

Gastric cancer (GC) represents the fourth most frequently occurring malignancy and the third leading death cause in relation to cancers across the globe^1^. It has become a significant social burden worthy of consideration, which is accompanied by relatively low rate (<10%) of diagnosis at early stage^2^. High morbidity and mortality have been reported for GC^3^, which is a heterogeneous tumor with variable environmental pathogenesis and pathways of tumorigenesis^4^. Notably, prior evidence has pointed out that autophagy is a promising target for antineoplastic strategy, due to its association with occurrence, cellular activities, and development^5,6^. It is interesting that noncoding RNAs (ncRNAs), such as microRNAs (miRNAs) and long noncoding RNAs (lncRNAs), have been suggested to modulate genes involved in the development of variable malignancies including GC^7^.

It is interesting to note that Krüppel-like factor 5 (KLF5) represents a zinc-finger transcription factor, which has a significant role to play in modulating cell behaviors during oncogenesis^8^. Prior evidence has identified a modulatory effect of KLF5 in the proliferation, malignant phenotypes of GC cells, and it may accelerate tumorigenesis in a tissue-specific manner by restarting early development programs^9–11^. As a transcriptional activator, KLF5 can directly bind to a specific recognition motif in the target gene promoter to activate transcription of its downstream targets, including ncRNAs^12^. Notably, lncRNA DANCR (ANCR) has been extensively studied due to its role as a ceRNA to sponge miRNAs and repress their expression levels, exerting an oncogenic effect in lung adenocarcinoma^13^, osteosarcoma^14^, and bladder cancer^15^. Interestingly, evidence exists demonstrating the downregulation and tumor-suppressive effect of miRNA-194 (miR-194) in GC^16^. Therefore, we speculated that DANCR can act as a ceRNA sponge to modulate miR-194 and inhibit its expression.

Furthermore, the in silico prediction of the present study identified the binding of miR-194 to AKT2. A prior study also indicated that miR-194 bound to AKT2 and thwarted the colorectal carcinogenesis^17^, the effect of miR-194 in GC may be realized through modulation through AKT2. As one of the members of the Akt family, AKT2 is abnormally highly expressed in GC tissues and cells, and has a profound association with tumorigenesis^18^. Therefore, we performed this study to test the hypothesis that the KLF5/DANCR/miR-194/AKT2 axis has a regulatory role to play in the progression of GC.

## Results

### KLF5 is highly expressed in GC and activates DANCR transcription to elevate its expression

**Fig. 1:**
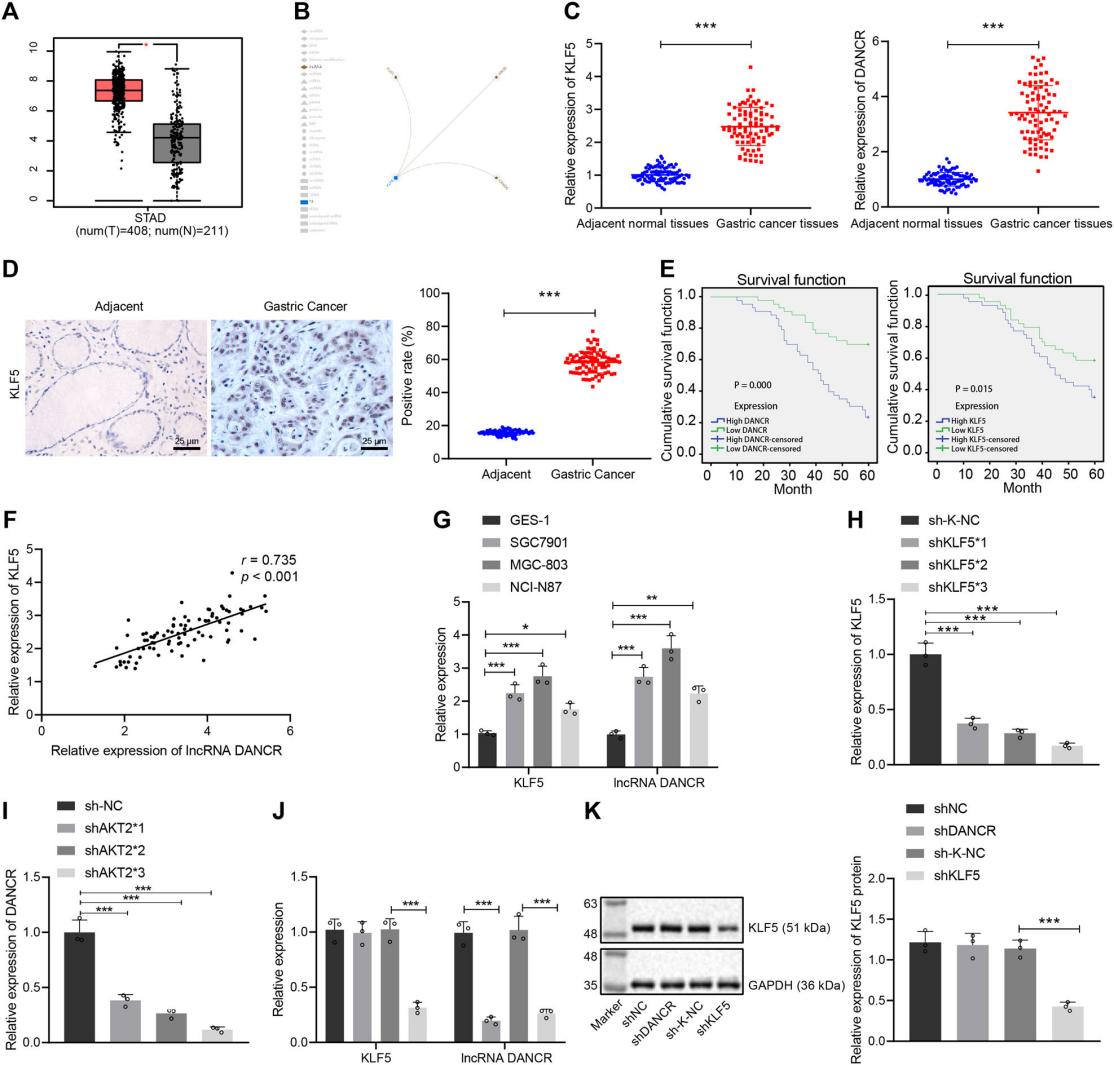
KLF5 is highly expressed in GC tissues and activates DANCR transcription to elevate its expression. **A** KLF5 expression boxplot of GC samples and normal samples in TCGA database. The abscissa represents the sample type, T represents the GC sample, N represents the normal sample, and the ordinate represents the sample expression; **B** RNAInter analysis (http://www.rna-society.org/rnainter/) to predict the lncRNAs targeted by KLF5; **C** qRT-PCR to determine the expression of KLF5 and DANCR in GC tissues and adjacent normal tissues (*n* = 86); **D** immunohistochemistry (400×) to detect the expression of KLF5 in GC tissues and adjacent normal tissues (*n* = 86); **E** Kaplan–Meier method for survival analysis; **F** correlation analysis between KLF5 expression and DANCR expression; **G** qRT-PCR screening of cell lines with the highest expression of KLF5 and DANCR; **H**, **I** qRT-PCR to assess the silencing efficiency of KLF5 and DANCR; **J** qRT-PCR measurement of KLF5 and DANCR expression in transfected cells of each group; **K** western blot analysis of KLF5 protein expression in transfected cells of each group. *p < 0.05, ***p < 0.0001. The measurement data are summarized by mean ± standard deviation. Data in **A**–**D** were analyzed by paired *t* test. Data in **G**–**K** were analyzed by one-way ANOVA with Tukey post hoc test. Pearson coefficient was employed to evaluate the correlation between KLF5 and DANCR. Kaplan–Meier method (log-rank test) was used for survival analysis in response to high or low expression of KLF5 and DANCR. Cellular experiment was repeated three times.

By querying the GEPIA database, we verified that KLF5 was highly expressed in the GC samples of the TCGA database (Fig. 1A). We predicted the lncRNAs targeted by KLF5 through the biological prediction website RNAInter and found that KLF5 regulated three lncRNAs: TUG1, ANCR (DANCR), and CRNDE (Fig. 1B). First, we verified through qRT-PCR the upregulated expression of KLF5 and DANCR in GC tissues (*p* < 0.001, Fig. 1C). In addition, immunohistochemical analysis found that the positive expression of KLF5 was brownish yellow, and the expression of KLF5 in GC tissue was upregulated (*p* < 0.001, Fig. 1D). Furthermore, 86 cases of GC were analyzed for correlation between KLF5 expression and clinicopathological characteristics, and found that KLF5 expression was not correlated with the patient’s sex and age (*p* > 0.05) but was related to the tumor size, TNM stage, invasion depth, and lymph node metastasis (*p* < 0.05) (Supplementary Table 1). Moreover, the overall survival (OS) of GC patients with low expression of KLF5 and DANCR was found to be higher than those with high expression (*p* < 0.001, Fig. 1E). In summary, KLF5 and DANCR were abnormally highly expressed in GC tissues, and the high expression of KLF5 and DANCR was associated with poor survival.

According to the characteristics of KLF5 that may restart the early development program, we speculated that KLF5 may activate the transcription of DANCR. First, the Pearson correlation analysis showed that KLF5 levels were positively correlated with DANCR expression (*p* < 0.05, Fig. 1F). We further verified the above speculation by using cell experiments and found that relative to normal gastric epithelial cells (GES-1), KLF5 and DANCR were highly expressed in GC cell lines, while the expression of KLF5 and DANCR was the highest in the MGC-803 cell line, which was selected for subsequent experiments (Fig. 1G). We transduced shNC, shDANCR, sh-K-NC, and shKLF5 plasmids on MGC-803 cells, and used qRT-PCR to evaluate the silencing efficiency. The expression of DANCR was the lowest in the presence of shDANCR#3, and the expression of KLF5 in the presence of shKLF5*3 was the lowest (*p* < 0.001, Fig. 1H, I). Therefore, shDANCR#3 and shKLF5*3 sequences were selected for subsequent experiments. In addition, the effect of silencing DANCR on KLF5 expression was not obvious (*p* > 0.05), while silencing KLF5 caused a decrease in DANCR expression (*p* < 0.001, Fig. 1J, K). In summary, KLF5 activated DANCR transcription in the promoter region and augments the increase of DANCR expression, thereby affecting the development of GC. Conversely, the knockdown of KLF5 downregulated the expression of DANCR.

### AKT2 is a target of miR-194

**Fig. 2:**
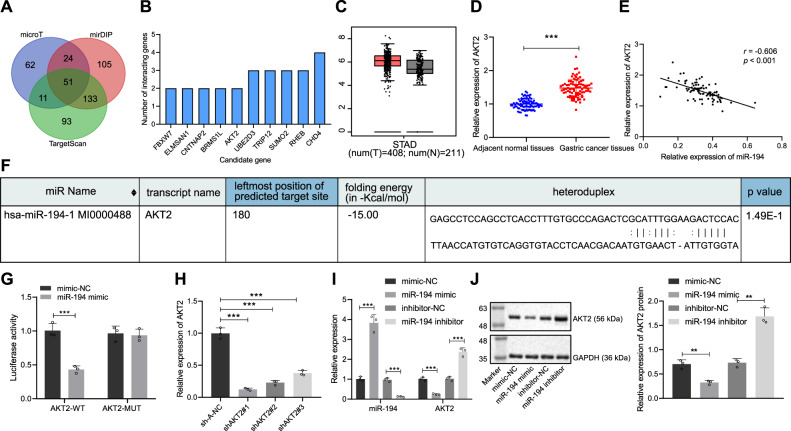
AKT2 is a target of miR-194. **A** Venn diagram (http://bioinformatics.psb.ugent.be/webtools/Venn/) of databases of microT (http://diana.imis.athena-innovation.gr/DianaTools/index.php?r=microT_CDS/), mirDIP (http://ophid.utoronto.ca/mirDIP/), and TargetScan (http://www.targetscan.org/vert_72/) to predict target genes of miR-194; **B** correlation and interaction analysis of target genes through STRING website (https://string-db.org/). The abscissa indicates the number of interacting genes, and the ordinate indicates the names of candidate genes; **C** AKT2 expression in GC and normal samples in the TCGA database. The abscissa represents the sample type, T represents the GC sample, N represents the normal sample, and the ordinate represents the sample expression level; **D** qRT-PCR to determine the expression of AKT2 mRNA in GC tissues and adjacent normal tissues; **E** Correlation analysis of miR-194 expression and AKT2 level; **F** Prediction of the binding site between AKT2 and miR-194 through the RNA22 website (https://cm.jefferson.edu/rna22/); **G** Dual luciferase assay to verify the targeting relationship between AKT2 and miR-194; **H** qRT-PCR to measure the silencing efficiency of AKT2; **I** qRT-PCR to determine miR-194 and AKT2 expression in each group of transfected cells; **J** western blot assay to determine the protein expression of AKT2 in transfected cells of each group. *p < 0.05, **p < 0.00, ***p < 0.0001. The measurement data are summarized by mean ± standard deviation. Data in **C**, **D** were analyzed by paired *t* test. Data in **H**–**J** were analyzed by one-way ANOVA with Tukey post hoc test. Data in **G** were analyzed by two-way ANOVA with Tukey post hoc test. Pearson coefficient was employed to evaluate the correlation between AKT2 and miR-194. Cellular experiment was repeated three times.

Next, we predicted the target genes of miR-194 through the databases of microT, mirDIP, and TargetScan, and applied the Venn tool to intersect the target genes, which screened out 51 target genes that may have binding sites with miR-194 (Fig. 2A). Through STRING analysis of 51 target genes, ten genes at the core position were selected as candidate genes (Fig. 2B). GEPIA was employed to verify the expression of AKT2 in GC samples in TCGA, confirming the high expression of AKT2 in GC (Fig. 2C). In addition, the expression of AKT2 mRNA in GC tissue was increased relative to adjacent normal tissues (*p* < 0.001, Fig. 2D). Pearson correlation analysis showed that miR-194 level was negatively correlated with AKT2 expression (*p* < 0.001, Fig. 2E). We found the presence of the binding site between AKT2 and miR-194 through the RNA22 website (Fig. 2F). Meanwhile, the dual luciferase reporter gene assay verified that, versus the mimic-NC group, the luciferase activity for the co-transfection of miR-194 mimic and AKT2-WT decreased (*p* < 0.001), while the luciferase activity of AKT2-MUT co-transfected with miR-194 mimic was not significantly different (*p* > 0.05), indicating that miR-194 can specifically bind to the AKT2 gene (Fig. 2G). In addition, qRT-PCR detection found that the shAKT2#1 sequence resulted in the lowest expression of AKT2 (*p* < 0.001) (Fig. 2H), so the shAKT2#1 sequence was selected for subsequent experiments. Overexpression of miR-194 repressed AKT2 mRNA (*p* < 0.01) and protein (*p* < 0.001) expression, while inhibition of miR-194 promoted AKT2 expression (Fig. 2I, J). In summary, AKT2 was highly expressed in GC, and AKT2 was the target gene of miR-194. miR-194 can target and inhibit AKT2 expression.

### Overexpression of miR-194 promotes GC cell autophagy by targeting AKT2

**Fig. 3:**
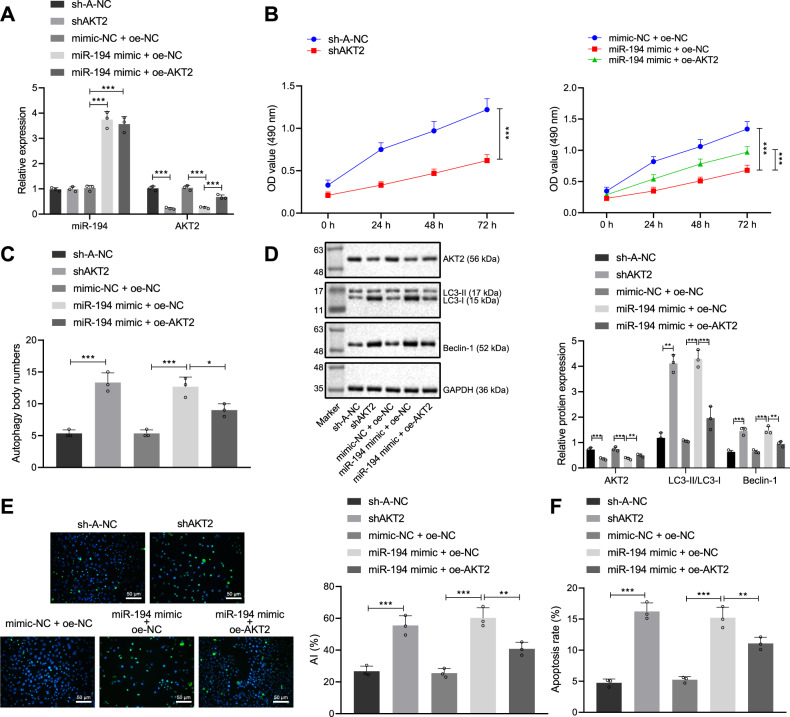
Overexpression of miR-194 promotes GC cell autophagy by targeting AKT2. **A** qRT-PCR to measure the expression of miR-194 and AKT2 in transfected cells; **B** MTT assay to assess the viability of transfected cells (Asterisk (*) signs indicate the *p* values for the entire difference between the curves); **C** quantitative analysis of autophagy of transfected cells under observation of transmission electron microscope; **D** western blot assay to measure the expression of AKT2 and autophagy-related proteins (LC3-I, LC3-II, and Beclin-1) in transfected cells; **E** TUNEL assay to detect TUNEL-positive apoptotic cells (200×); **F** flow cytometric analysis for the apoptosis rate of transfected cells; *p < 0.05, **p < 0.00, ***p < 0.0001. The measurement data are summarized by mean ± standard deviation. Data in **A**–**F** were analyzed by one-way ANOVA with Tukey post hoc test. Statistical analysis in relation to time-based measurements in **B** was realized using repeated measures ANOVA, followed by a Bonferroni’s post hoc test. Cellular experiment was repeated three times.

To explain the effect of the miR-194/AKT2 axis on GC biological function, we performed a functional assay. It was found that the shAKT2 plasmid was successfully transduced into MGC-803 cells (*p* < 0.001). The effect of silencing AKT2 on miR-194 levels was not obvious (*p* > 0.05). miR-194 overexpression repressed the expression of AKT2 gene and OE-AKT2 plasmid (*p* < 0.001) (Fig. 3A). MTT assay revealed that silencing AKT2 or overexpressing miR-194 repressed cell viability. Relative to miR-194 mimic alone, simultaneous overexpression of miR-194 and AKT2 can increase cell viability (*p* < 0.001, Fig. 3B). Transmission electron microscopy (TEM) revealed that silencing AKT2 or overexpressing miR-194 promoted cell autophagy, and elevated the number of autophagosomes (*p* < 0.001). Relative to miR-194 mimic alone, simultaneous overexpression of miR-194 and AKT2 can inhibit cell autophagy and reduce the number of autophagosomes (*p* < 0.05, Fig. 3C, Supplementary Fig 1A). The conversion of LC3-I to LC3-II during autophagy results in a decrease in LC3-I levels and an increase in LC3-II/LC3-I ratio. Silencing AKT2 or overexpressing miR-194 elevated the protein expression of Beclin-1 and enhanced the LC3-II/LC3-I ratio. Relative to miR-194 mimic alone, simultaneous overexpression of miR-194 and AKT2 repressed the protein expression of Beclin-1 and reduced the LC3-II/LC3-I ratio (*p* < 0.001 or *p* < 0.01) (Fig. 3D). TUNEL assay revealed that silencing AKT2 or overexpressing miR-194 led to an increase in TUNEL-positive cells (*p* < 0.001). Relative to miR-194 mimic alone, simultaneous overexpression of miR-194 and AKT2 repressed apoptosis and reduced TUNEL-positive cells (*p* < 0.01, Fig. 3E). Flow cytometric detection demonstrated that silencing AKT2 or overexpressing miR-194 accelerated apoptosis (*p* < 0.001). Versus miR-194 mimic alone, simultaneous overexpression of miR-194 and AKT2 repressed apoptosis (*p* < 0.01, Fig. 3F, Supplementary Fig. 1B). Therefore, AKT2 did not affect the expression of upstream regulator miR-194. Silencing AKT2 activated GC cell autophagy, and overexpression of AKT2 repressed GC cell autophagy. Upregulation of miR-194 can promote GC cell autophagy and prevent GC development by targeting AKT2.

### DANCR acts as a ceRNA to sponge miR-194, thereby downregulating its expression in GC

**Fig. 4:**
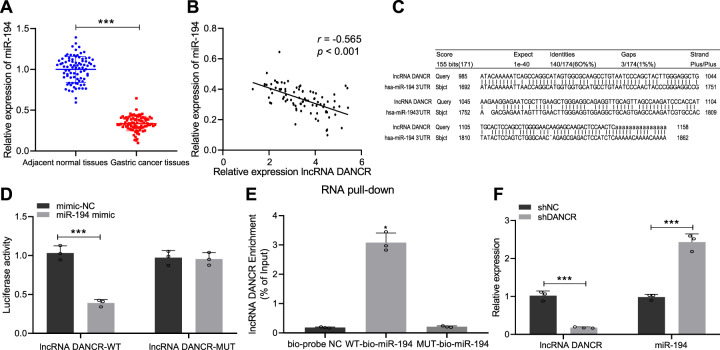
DANCR acts as a ceRNA to sponge miR-194, thereby downregulating its expression in GC. **A** qRT-PCR to measure the expression of miR-194 in GC tissue and adjacent normal tissues (*n* = 86); **B** correlation analysis between miR-194 expression and DANCR level; **C** the binding site between DANCR and miR-194 predicted through the Blast website (https://blast.ncbi.nlm.nih.gov/Blast.cgi?PAGE=MegaBlast&PROGRAM=blastn&BLAST_PROGRAMS=megaBlast&PAGE_TYPE=BlastSearch&BLAST_SPEC=blast2seq&DATABASE=n/a); **D** dual luciferase report assay to verify the targeting relationship between DANCR and miR-194; **E** RNA pull-down assay to verify that DANCR specifically sponged miR-194; **F** qRT-PCR to measure the expression of DANCR and miR-194 in transfected cells. *p* < 0.05, ***p < 0.0001. The measurement data are summarized by mean ± standard deviation. Data in **A** were analyzed by paired *t* test, while those in **F** were analyzed by unpaired *t* test. Data in **E** were analyzed by one-way ANOVA with Tukey post hoc test. Pearson coefficient was employed to evaluate the correlation between miR-194 and lncRNA DANCR. Cellular experiment was repeated three times.

We speculated that DANCR functioned as a ceRNA to sponge miR-194 and repressed its expression. The following experiments were conducted to verify this speculation. We found that the expression of miR-194 in GC tissue was reduced versus the adjacent normal tissues (*p* < 0.001, Fig. 4A). The level of miR-194 was revealed to be negatively correlated with DANCR expression (*p* < 0.001, Fig. 4B), suggesting that miR-194 was inversely regulated by DANCR. Through the Blast website analysis, we found the presence of binding sites between DANCR and miR-194 (Fig. 4C). Moreover, the dual luciferase reporter gene assay verified that, versus mimic-NC, co-transfection of DANCR-WT and miR-194 mimic resulted in decreased luciferase activity (*p* < 0.001), while co-transfection of DANCR-MUT and miR-194 mimic showed no obvious effects (*p* > 0.05), indicating that miR-194 can specifically bind to the lncRNA DANCR (Fig. 4D). Further, versus the bio-probe negative control (NC) control group, WT-bio-miR-194 promoted DANCR enrichment (*p* < 0.05), while MUT-bio-miR-194 led to no significant difference in DANCR levels (*p* > 0.05), suggesting that DANCR can specifically sponge miR-194 (Fig. 4E). Finally, we interfered with the expression of DANCR in MGC-803 cells, and qRT-PCR detection found that silencing DANCR resulted in an upregulation of miR-194 expression (*p* < 0.001, Fig. 4F). Therefore, miR-194 was lowly expressed in GC, and DANCR repressed miR-194 expression in GC through functioning as a ceRNA. Knockdown of DANCR can increase the level of miR-194.

### DANCR knockdown upregulates miR-194 expression, thereby accelerating GC cell autophagy

**Fig. 5:**
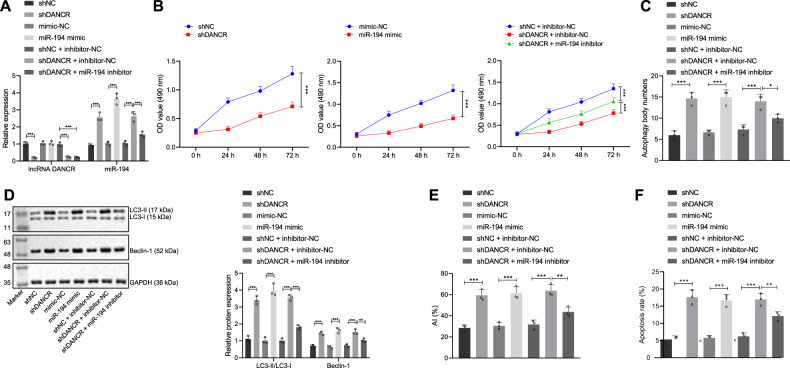
DANCR knockdown upregulates miR-194 expression, thereby accelerating GC cell autophagy. **A** qRT-PCR to measure the mRNA expression of DANCR and miR-194 in transfected cells; **B** MTT assay to detect the viability of transfected cells; **C** Quantitative analysis of autophagy of transfected cells under the observation of transmission electron microscope; **D** western blot assay to measure the expression of autophagy-related proteins (LC3-I, LC3-II, and Beclin-1); **E** TUNEL assay to assess TUNEL-positive apoptotic cells; **F** Flow cytometric detection of apoptosis rate of the transfected cells. *p < 0.05, **p < 0.00, ***p < 0.0001. The measurement data are summarized by mean ± standard deviation. Data in **A**–**F** were analyzed by one-way ANOVA with Tukey post hoc test. Statistical analysis in relation to time-based measurements in **B** was realized using repeated measures ANOVA, followed by a Bonferroni’s post hoc test. Cellular experiment was repeated three times.

In order to probe the effect of the DANCR/miR-194 axis on viability, autophagy and apoptosis of GC cells, we transduced shNC, shDANCR, mimic-NC, miR-194 mimic, shNC + inhibitor-NC, shDANCR + inhibitor-NC, and shDANCR + miR-194 inhibitor plasmids into MGC-803 cells. The data demonstrated that shDANCR knocked down DANCR expression in GC cells and miR-194 mimic overexpressed miR-194 in GC cells. The effect of miR-194 overexpression on the expression of DANCR was not obvious (*p* > 0.05), while silencing DANCR upregulated the expression of miR-194 (*p* < 0.05). Meanwhile, in the presence of shDANCR, miR-194 inhibitor repressed miR-194 expression in GC cells (*p* < 0.001, Fig. 5A). MTT assay revealed that silencing DANCR or overexpressing miR-194 repressed cell viability (*p* < 0.001). Relative to shDANCR alone, simultaneous inhibition of DANCR and miR-194 promoted cell viability (*p* < 0.001, Fig. 5B). Transmission electron microscopic observation displayed that silencing DANCR or overexpressing miR-194 augmented autophagy and increased the number of autophagosomes. Relative to shDANCR alone, simultaneous inhibition of DANCR and miR-194 repressed autophagy and reduced the number of autophagosomes (*p* < 0.05, Fig. 5C, Supplementary Fig. 1C). Silencing DANCR or overexpressing miR-194 accelerated the protein expression of Beclin-1 and the LC3-II/LC3-I ratio (*p* < 0.001). Relative to shDANCR alone, simultaneous inhibition of DANCR and miR-194 halted the protein expression of Beclin-1 and the LC3-II/LC3-I ratio (*p* < 0.001 or *p* < 0.01, Fig. 5D). TUNEL assay found that the knockdown of DANCR or overexpression of miR-194 elevated TUNEL-positive apoptotic cells (*p* < 0.001). Relative to shDANCR alone, simultaneous inhibition of DANCR and miR-194 impeded apoptosis and reduced TUNEL-positive apoptotic cells (*p* < 0.01, Fig. 5E). Furthermore, the knockdown of DANCR or the overexpression of miR-194 promoted apoptosis (*p* < 0.001). Relative to shDANCR alone, simultaneous inhibition of DANCR and miR-194 impeded cell apoptosis (*p* < 0.01, Fig. 5F, Supplementary Fig. 1D). In summary, miR-194 inhibition accelerated GC cell viability and repressed its autophagy and apoptosis. Knockdown of DANCR upregulated miR-194 to inhibit GC cell viability and promote its autophagy and apoptosis, preventing GC development.

### KLF5 knockdown promotes GC cell autophagy by regulating the DANCR/miR-194/AKT2 axis

**Fig. 6:**
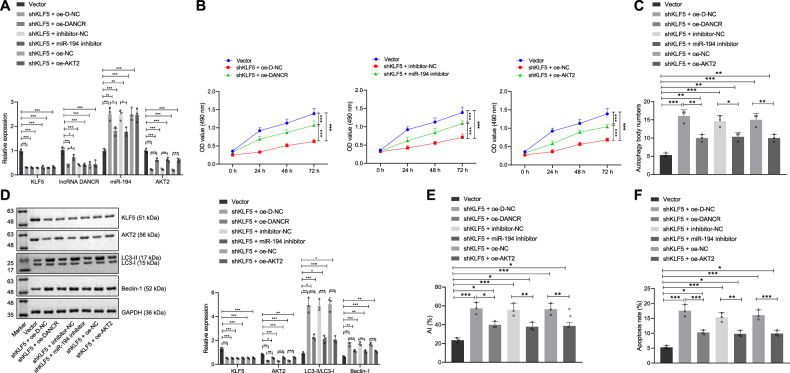
KLF5 knockdown promotes GC cell autophagy by regulating the DANCR/miR-194/AKT2 axis. **A** qRT-PCR to measure the expression of KLF5, DANCR, miR-194, and AKT2 in transfected cells; **B** MTT assay to detect the viability of transfected cells; **C** quantitative analysis of autophagy of transfected cells under the observation of transmission electron microscope; **D** western blot assay to measure the expression of KLF5, AKT2, and autophagy-related proteins (LC3-I, LC3-II, and Beclin-1) in transfected cells; **E** TUNEL assay to detect TUNEL-positive apoptotic cells; **F** flow cytometry to evaluate the apoptosis rate of transfected cells. *p < 0.05, **p < 0.00, ***p < 0.0001. The measurement data are summarized by mean ± standard deviation. Data in **A**, **C**–**F** were analyzed by one-way ANOVA with Tukey post hoc test. Statistical analysis in relation to time-based measurements in **B** was realized using repeated measures ANOVA, followed by a Bonferroni’s post hoc test. Cellular experiment was repeated three times.

In order to further clarify the effect of the KLF5/DANCR/miR-194/AKT2 axis on the biological function of GC, we transduced plasmids of Vector, shKLF5 + OE-D-NC, shKLF5 + OE-DANCR, shKLF5 + inhibitor-NC, shKLF5 + miR-194 inhibitor, shKLF5 + OE-NC, and shKLF5 + OE-AKT2 to MGC-803 cells. It was found that silencing KLF5 inhibited the expression of DANCR, thereby upregulating the level of miR-194 and inhibiting the expression of AKT2 (*p* < 0.001). The effect of overexpression of DANCR on the level of KLF5 was not obvious (*p* > 0.05), but downregulated miR-194 expression (*p* < 0.05). Downregulation of miR-194 exerted no obvious effects on KLF5 and DANCR levels (*p* > 0.05) but diminished AKT2 expression (*p* < 0.001). The effect of overexpression of AKT2 on the levels of KLF5, DANCR, and miR-194 was not significant (*p* > 0.05) (Fig. 6A). MTT assay revealed that the rest plasmids inhibited cell viability vs. vector (*p* < 0.001). Relative to KLF5 knockdown, overexpressed DANCR, inhibited miR-194 or overexpressed AKT2 all accelerated cell viability (*p* < 0.001) (Fig. 6B). Transmission electron microscopic observation showed that relative to vector, the remaining plasmids activated cell autophagy and increased the number of autophagosomes (*p* < 0.001 or *p* < 0.01). Relative to KLF5 knockdown, overexpressed DANCR, downregulated miR-194 or overexpressed AKT2 all inhibited autophagy and reduced the number of autophagosomes (*p* < 0.01 or *p* < 0.05) (Fig. 6C, Supplementary Fig. 1E). Western blot assay revealed that compared with vector, the remaining plasmids decreased the protein expression of KLF5 and AKT2 (*p* < 0.001, *p* < 0.01, or *p* < 0.05), increased the protein expression of Beclin-1 (*p* < 0.001 or *p* < 0.01), and promoted the LC3-II/LC3-I ratio (*p* < 0.001 or *p* < 0.05). Relative to KLF5 knockdown, the effect of overexpression of DANCR, downregulation of miR-194 or overexpression of AKT2 on the protein expression of KLF5 was not obvious (*p* > 0.05) but promoted the expression of AKT2 protein (*p* < 0.001 or *p* < 0.01), while inhibiting the protein expression of Beclin-1 (*p* < 0.001) and the LC3-II/LC3-I ratio (*p* < 0.001) (Fig. 6D). TUNEL assay found that compared with vector, the remaining plasmids increased TUNEL-positive apoptotic cells (*p* < 0.001 or *p* < 0.05). In comparison with KLF5 knockdown, overexpressed DANCR, downregulated miR-194 or overexpressed AKT2 all inhibited apoptosis and reduced TUNEL-positive apoptotic cells (*p* < 0.01 or *p* < 0.05, Fig. 6E). Flow cytometry revealed that the remaining plasmids increased apoptosis compared to the vector group (*p* < 0.001 or *p* < 0.05). Relative to KLF5 knockdown, overexpressed DANCR, downregulated miR-194, or overexpressed AKT2 all inhibited apoptosis (*p* < 0.001 or *p* < 0.01, Fig. 6F, Supplementary Fig. 1F). Therefore, KLF5 knockdown downregulated the expression of DANCR and promoted the level of miR-194, thereby inhibiting the expression of AKT2 to promote the autophagy and apoptosis of GC cells, and to repress the viability of GC cells, which ultimately prevents the development of GC.

### KLF5 knockdown inhibits tumor growth in vivo by regulating the DANCR/miR-194/AKT2 axis

**Fig. 7:**
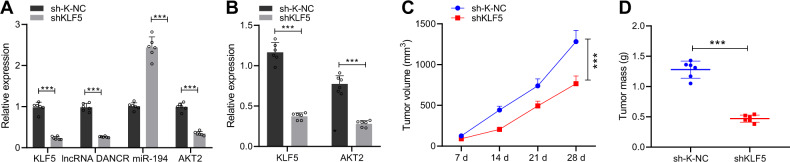
KLF5 knockdown inhibits tumor growth in vivo by regulating the DANCR/miR-194/AKT2 axis. **A** qRT-PCR to determine the expression of KLF5, DANCR, miR-194, and AKT2 in nude mouse tumor tissue; **B** western blot assay to measure the protein expression of KLF5 and AKT2 in nude mouse tumor tissue; **C** quantitative analysis of tumor volume growth in nude mice; **D** quantitative analysis of tumor weight in nude mice; *p < 0.05, **p < 0.00, ***p < 0.0001. The measurement data are summarized by mean ± standard deviation. Data in **A**, **B**, **D** were analyzed by unpaired *t* test. Statistical analysis in relation to time-based measurements in **C** was realized using repeated measures ANOVA, followed by a Bonferroni’s post hoc test.

We conducted in vivo experiments to investigate whether silencing KLF5 can inhibit the growth of transplanted tumors in nude mice by regulating the DANCR/miR-194/AKT2 axis. We injected the stably transfected cells subcutaneously in nude mice to construct a subcutaneous xenograft model. The experimental results showed that all tumors at the inoculation sites had visible tumor growth, presenting with small subcutaneous nodules at the inoculation sites, which were initially elliptical, and gradually became irregular and irregularly lobulated on the surface. The results of qRT-PCR showed that silencing KLF5 downregulated DANCR and increased the level of miR-194, thereby inhibiting the expression of AKT2 (*p* < 0.001, Fig. 7A). Further, silencing KLF5 inhibited AKT2 protein expression (*p* < 0.001, Fig. 7B). We measured nude mouse tumors for 4 consecutive weeks to draw growth curves, and the data illustrated that silencing KLF5 can inhibit tumor growth (*p* < 0.001, Fig. 7C) and reduce tumor weight (*p* < 0.001, Fig. 7D). In summary, silencing KLF5 increased miR-194 levels by down-regulating DANCR expression, thereby inhibiting AKT2 expression, which ultimately impeded GC cell growth in vivo.

## Discussion

GC represents one of the most common malignancies worldwide, characterized by alarming mortality and increasing prevalence^19^. Accumulating evidence has highlighted the involvement of lncRNAs in carcinogenesis *via* interaction with miRNA-mRNA, including GC progression^20^. Efforts have been made to explore the modulatory role of aberrant lncRNA-miRNA-mRNA network, and the mechanism of lncRNAs has been attributed to the regulation of protein-coding gene expression through sponging miRNA^21^. Here in this study, we introduce the effects of KLF5 in GC, emphasizing the mechanisms of KLF5 associated with the DANCR/miR-194/AKT2 axis.

The experimental observations uncovered that KLF5 and DANCR were highly expressed in GC tissues and cells, which may exercise a carcinogenic effect. More importantly, KLF5 was revealed to activate DANCR transcription to elevate its expression. KLF5 is a pivotal transcription factor of variable morphogenetic programs, and activity of KLF5 in gastrointestinal tract has been observed^22^. The upregulation of KLF5 has been previously demonstrated in GC tissues and cells, which resulted in tumor aggressiveness^23^. Evidence exists supporting the pro-tumorigenic effects of DANCR overexpression which occurs in GC^24^. Therefore, the tumor-supporting properties of KLF5 in GC were achieved through activating DANCR transcription.

Next, we investigated the specific function of DANCR in GC cells, and found that DANCR functioned as a ceRNA to sponge miR-194, thereby downregulating its expression in GC. Previous evidence has suggested the tumor-initiating activities of DANCR in CRC, which was realized through sponging miR-577 as a ceRNA^25^. Moreover, the oncogenic role of DANCR in non-small cell lung cancer has been documented to be associated with its modulation of the tumor suppressor miR-758-3p as a ceRNA^26^. Similarly, we also identified the anti-tumor effect of miR-194 in GC, and overexpression of miR-194 augmented GC cell autophagy, thus impeding the progression of GC. Furthermore, knockdown of DANCR upregulated miR-194 expression, thereby accelerating GC cell autophagy and halting GC development. In the human gastrointestinal tract, miR-194 is specifically expressed and might exert a tumor-suppressive effect in GC^27^. In addition, a prior study has suggested the potential value of miR-194 in early detection and prognostic significance of GC, due to its confirmed modulatory effect on malignant phenotype of GC cells^28^. The study of Li et al. has indicated a post-transcriptional regulatory mechanism of the target gene FoxM1 of miR-194 for its tumor-inhibiting activities in GC^29^.

Further mechanistic investigation unraveled the targeting relationship of miR-194 and AKT2 determined based on bioinformatics prediction and luciferase activity assay. Upregulation of miR-194 augmented GC cell autophagy, apoptosis and impeded its viability through repressing AKT2 expression. Previous evidence has illuminated that AKT2 was targeted and negatively mediated by miR-194, and the upstream lncRNA SOX2OT served as a miR-194 sponge to upregulate AKT2, intensifying tumor growth and metastasis of GC^30^. Furthermore, the Akt pathway is one of the most common activation pathways in human cancers, and it connects carcinogenic receptors with many pro-survival cytokines^31^. In addition, AKT2 was associated with tumorigenesis of GC, and AKT2 knockdown can inhibit the proliferative potential of tumor cells^32^. Our data have validated that KLF5 knockdown accelerated GC cell autophagy in vitro and repressed tumor growth in vivo by regulating the DANCR/miR-194/AKT2 axis.

**Fig. 8:**
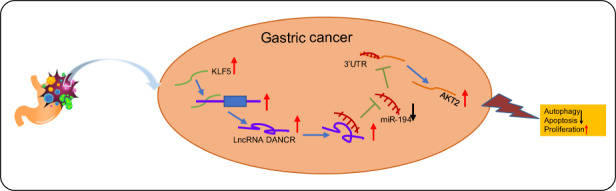
Mechanism graph of the regulatory network and oncogenic function of KLF5 in gastric cancer. KLF5 activates the transcription of DANCR in the promoter region and elevates DANCR expression. DANCR acts as a miR-194 sponge to repress its expression. MiR-194 targets and inhibits AKT2 expression. Therefore, KLF5 promotes the malignant phenotypes of gastric cancer through activation of lncRNA DANCR and impairs miR-194-dependent AKT2 inhibition.

In the present work, we verified the oncogenic function of KLF5 (Fig. 8) in curbing apoptosis and autophagy of GC cells through a series of functional experiments both in vitro and in vivo. Mechanistically, KLF5 knockdown prevents the activation of lncRNA DANCR and upregulates miR-194-dependent AKT2 inhibition. As we further elucidate the epigenetic regulatory mechanisms underlying GC, there is great potential for translational applications for GC patients.

## Methods

### Ethics statement

This study was approved by the ethics committee of the Hospital Afiliated 5 to Nantong University (Taizhou People’s Hospital), and all patients provided written informed consent. All procedures were performed in accordance with the Helsinki Declaration. Animal experiments were performed in accordance with *Guide for the Care and Use of Laboratory Animals* published by the National Institutes of Health, and approved by the Animal Ethics Committee of the Hospital Afiliated 5 to Nantong University (Taizhou People’s Hospital).

### Patients

The specimens of 86 GC patients diagnosed between January 2010 and January 2014 in the Hospital Afiliated 5 to Nantong University (Taizhou People’s Hospital) were selected as the study subjects, including 55 males and 31 females, with a mean age of 59.85 ± 6.88 years. The patients were included in this study if (1) they had not received radiochemotherapy or other treatment before surgery, and the clinical data were complete; (2) the age was 3–70 years old; (3) they had no other malignant tumors, no mental abnormalities, or unconscious disorders. The following patients were excluded from this study: (1) patients aged <3 or >70 years; (2) patients during pregnancy or lactation; (3) patients with other severe systemic diseases or malignant tumors. Adjacent normal tissue samples (>5 cm from the outer edge of the tumor) from the same patient were taken as controls (adjacent group). All sections were confirmed pathologically by two experienced pathologists. A portion of the tissue sample was immediately stored in liquid nitrogen. The other part of the tissue sample was fixed with 10% formalin and embedded in paraffin for preservation after routine dehydration. These patients were followed up for 60 months. During the follow-up period, the death of the patient was taken as the end point of the event. If the event did not occur, the final follow-up time was taken as the end point. The time interval from the date of surgery to the date of death is defined as OS.

### Immunohistochemistry

Paraffin specimens of tissues to be tested were sliced to a thickness of 4 μm, and dewaxed and hydrated. The primary antibody rabbit anti-KLF5 (ab137676, 1:100) and the secondary antibody IgG (ab205718, 1:2000) were all purchased from Abcam (Cambridge, UK). Saline instead of primary antibody was employed as a NC. Five fields were randomly selected under the microscope to count positively stained cells. The density of positive cells can also be semi-quantitatively analyzed based on the percentage of positive cells^33,34^. According to the staining situation, the number of KLF5 positive cells <25% was regarded as negative, and the number of positive cells >25% as positive.

### Cell lines

Three GC cell lines (SGC7901, MGC-803, and NCI-N87) and one normal GES-1 were purchased from the Cell Bank of the Chinese Academy of Sciences (Shanghai, China). The recovered cells were cultured in DMEM (12800017, Gibco) containing 10% fetal bovine serum (FBS, 26140079, Gibco) and 1% double antibody (5% CO_2_, 37 °C). The culture medium was changed every 24 h and passaged every 72 h. The cells were digested with 0.25% trypsin and gently pipetted into single-cell suspension. After routine passage, the cells in logarithmic growth phase were examined for the expression of KLF5 and DANCR by qRT-PCR. The cell line with the highest expression of KLF5 and DANCR was selected for subsequent experiments.

### Lentiviral production and transduction

Cells in the logarithmic growth phase (5 × 10^5^ cells/well) were seeded in six-well cell culture plates and transfected when cell confluence reached 60–80%, on the basis of the protocols of the lipofectamine 2000 kit (11668-027, Invitrogen). Serum-free RPMI 1640 medium was employed to dilute transfection sequences (GenePharma, Shanghai, China, final concentration = 50 nM) and lipofectamin 2000 (5 µL). The cells were transduced with lentiviral vectors containing shRNA (sh)KLF5, shDANCR, shAKT2, overexpression plasmid (OE)-AKT2, OE-DANCR, as well as miR-194 mimic and miR-194 inhibitor, along with corresponding NC sequences: shRNA NC of KLF5 (sh-K-NC), shNC, mimic-NC, inhibitor-NC, shRNA NC of AKT2 (sh-A-NC), OE-NC, empty vector, overexpression plasmid NC of DANCR (OE-D-NC). The interference efficiency of shRNAs (shDANCR#1, shDANCR#2, shDANCR#3, shKLF5*1, shKLF5*2, shKLF5*3, shAKT2#1, shAKT2#2, and shAKT2#3) (Supplementary Table 2) was evaluated by qRT-PCR to select the shRNA sequences with the lowest expression of KLF5, DANCR, and AKT2 for subsequent experiments. All shRNAs were first transduced into HEK293T cells (purchased from CoBioer Biosciences CO., LTD, Nanjing, China) for lentivirus packaging, and then GC cells were infected with lentivirus. The construction, sequencing identification, virus packaging, and titer detection of all plasmids and vector were performed by Shanghai GeneChem (Shanghai, China).

### RNA extraction and RT-qPCR

Total RNA from tissue homogenate or cells was extracted using RNeasy Mini Kit (Qiagen, Valencia, CA). Then, cDNA was obtained utilizing a reverse transcription kit (RR047A, Takara, Japan) and miRNA First Strand cDNA Synthesis kit (B532451-0020, Sangon Biotech., Shanghai, China). Sample loading was performed using SYBR^®^ Premix Ex Taq^TM^ II (Perfect Real Time) kit (DRR081, Takara, Japan). The samples were subjected to a real-time fluorescence quantitative PCR instrument (ABI 7500, ABI, Foster City, CA). The miRNA universal negative primer and the upstream primer of the U6 internal reference were provided in the miRNA First Strand cDNA Synthesis (Tailing Reaction) kit, and other primers were synthesized by Sangon Biotech (Supplementary Table 3). Using GAPDH or U6 as an internal reference, the relative quantification method (2^−^^ΔΔCt^) was employed to calculate the relative transcription level of the gene to be tested.

### Western blots

The total protein of cells or tissues was extracted using PMSF-containing RIPA lysis buffer (R0010, Solarbio, China) pre-cooled at 4 °C, followed by BCA kit (20201ES76, Yeasen, Shanghai, China) to determine the protein concentration. The proteins separated by polyacrylamide gel electrophoresis were transferred to PVDF membrane (P2438, Sigma) by wet transfer method, and the membrane was blocked with 5% BSA at room temperature for 1 h. The blots were probed with dilute primary antibody rabbit anti-KLF5 (ab137676, 1:100), AKT2 (ab32505, 1:3000), LC3B (ab51520, 1:3000, Abcam, UK), Beclin-1 (ab207612, 1:2000, Abcam, UK), and GAPDH (ab181602, 1:10000) overnight at 4 °C. Thereafter, the membrane was incubated with HRP-labeled goat anti-rabbit secondary IgG (ab6721, 1:5000) at room temperature for 1 h. The above antibodies were purchased from Abcam (Cambridge, UK). The blots were visualized using the enhanced chemiluminescence and quantified with Quantity One v4.6.2 software, with GAPDH as internal control. All blots were derived from the same experiment and were processed in parallel.

### MTT assay

Cells (5 × 10^6^–1 × 10^7^ per well) were seeded in 96-well plates with a volume of 200 μL per well. Each sample was repeated in six wells. After incubation for 48 h, 20 μL of 5 mg/mL MTT solution (A2776-1g, Shifeng Biotechnology, Shanghai, China) was added to each well for 4 h incubation. The culture medium was discarded and 150 μL of DMSO was added. After the cells were incubated for 0, 24, 48, and 72 h, the optical density of each well at a wavelength of 490 nm was assessed using an enzyme-linked immunoassay plate reader.

### Transmission electron microscopy (TEM)

The cells were fixed with 2.5% glutaraldehyde solution at 4 °C overnight, and then fixed with osmic acid at 4 °C for 1 h. The cells were then dehydrated with acetone, embedded and polymerized to prepare electron microscope sections. Then the sections were photographed and analyzed using a JEMI400 TEM at an acceleration voltage of 80 kv. The gold standard for testing whether cells undergo autophagy was to observe whether autophagosomes were produced in the cytoplasm under transmission electron microscope. The structure of a typical autophagosome is a spherical structure composed of a double-layer or multi-layer membrane, which encloses the substance to be degraded.

### TUNEL assay

The digested logarithmic growth phase cells were incubated with FBS-free RPMI 1640 medium for 12 h. After 24 h, the cells were fixed in 4% paraformaldehyde, permeabilized in 1% Triton X-100 solution and blocked in 3% H_2_O_2_. The color reaction was performed following the protocols of the in situ detection kit for apoptosis (11684817910, Solarbio, Beijing, China). The developed cells were stained with hematoxylin (PT001, Bogoo Biotechnology, Shanghai, China), immersed in 1% hydrochloric acid alcohol for hydrolyzation, dehydrated, permeabilized, and sealed. Stained cells were observed under a microscope and photographed. TUNEL-positive cells and total cells were counted, and the percentage of positive cells in total cells was calculated as the apoptosis index.

### Flow cytometry analysis of cell apoptosis

The cells, 48 h after transfection, were digested with 0.25% trypsin without EDTA, and the supernatant was discarded after centrifugation. The assay was performed on the basis of the protocols of Annexin-V-FITC Apoptosis Detection Kit (556547, Shuojia Biotechnology, Shanghai, China). The flow cytometer (BIO-RAD, Hercules, CA) was employed to evaluate cell apoptosis.

### Luciferase activity assay

The target site sequence (WT) and the sequence (MUT) after targeted mutation of the WT target site of DANCR and AKT2 mRNA 3′-UTR region were synthesized. Restriction enzymes were adopted to digest pmiR-RB-REPORT^TM^ plasmid (RiboBio, Guangzhou, China), and the artificially synthesized target gene fragments WT and MUT were inserted into pmiR-RB-REPORT^TM^ vector, respectively. The luciferase reporter plasmids WT and MUT that were sequenced correctly were used for subsequent transfection. The vectors containing MUT and WT were co-transfected into HEK293T cells with mimic-NC or miR-194 mimic, respectively. After 48 h of transfection, the cells were collected and lysed. The Renilla luciferase detection kit (YDJ2714, Shanghai Yuduo Biological Technology) was used to determine relative luciferase unit. With firefly luciferase as an internal reference, luciferase activities were analyzed using a dual luciferase report analysis system (Promega Co, Madison, WI).

### RNA pull-down assay

Cells were transfected with WT-bio-miR-194/MUT-bio-miR-194 labeled with 50 nM biotin, and bio-probe NC labeled with 50 nM biotin was used as a control. After 48 h, the cells were incubated in specific lysis buffer for 10 min. The lysate was incubated with M-280 streptavidin magnetic beads (S3762, Sigma) pre-coated with RNase-free BSA and yeast tRNA (TRNABAK-RO, Sigma) at 4 °C overnight. The bound RNA was purified by Trizol, and then qRT-PCR was adopted to identify the degree of enrichment of DANCR.

### Xenografts in nude mice

A total of 12 male BALB/c nude mice (J004, Better Biotechnology., Nanjing, China), aged 3–4 weeks old, and weighing 14–18 g, were raised under SPF conditions. The feeds used were all sterilized, the feeding temperature was 18–22 °C, and the humidity was 50–60%. Nude mice were randomly divided into two groups, six in each group. Stably transfected cell lines (sh-K-NC group and shKLF5 group) were digested with 0.25% trypsin, and the digestion was terminated with complete medium. The cell pellet was collected by centrifugation, and gently pipetted into a single cell suspension with saline. The cells were resuspended in normal saline (2 × 106 cells/50 μL), then mixed with 50 μL Matrigel (1:1), and inoculated into the axilla of nude mice. Nude mice were euthanized by injecting 40 mg/kg pentobarbital at the time points of 7, 14, 21, and 28 days, respectively, and xenograft tumors were collected to record weight and volume of tumors.

### Statistical analysis

Statistical analysis was performed using SPSS 22.0 statistical software (IBM Corp, Armonk, NY), with *p < 0.05, **p < 0.00, ***p < 0.0001 indicating statistical significance. Measurement data were expressed as mean ± standard deviation. The data of cancer tissues and adjacent normal tissues were analyzed by paired *t* test. The data comparison of other two groups was performed by unpaired *t* test. The comparison among multiple groups was analyzed by one-way analysis of variance (ANOVA) with Tukey post hoc test. Statistical analysis in relation to time-based measurements was realized using repeated measures ANOVA, followed by a Bonferroni’s post hoc test. Tukey or Bonferroni tests were used in the analysis of pairwise comparisons. This study only reported those that were statistically significant or mentioned in the text, and no other adjustments were made for multiple statistical testing. Pearson coefficient was employed for correlation analysis. Kaplan–Meier method (log-rank test) was employed to analyze the relationship between expression of KLF5 and DANCR in GC tissues and OS of patients. Enumeration data were presented as percentage and analyzed by Chi-square test.

### Reporting summary

Further information on research design is available in the Nature Research Reporting Summary linked to this article.

## Supplementary information

Supplementary Information

Reporting Summary

Supplementary Table 1

## Data Availability

Data available on request from the authors.
