# Supplementary material for: KLF5 activates lncRNA DANCR and inhibits cancer cell autophagy accelerating gastric cancer progression
Source: NPJ Genom Med. 2021 Sep 21;6:75. doi: 10.1038/s41525-021-00207-7 (PMC8455684; doi:10.1038/s41525-021-00207-7)
Supplement: Supplementary file 1 — Supplementary Information [file 41525_2021_207_MOESM1_ESM.pdf]

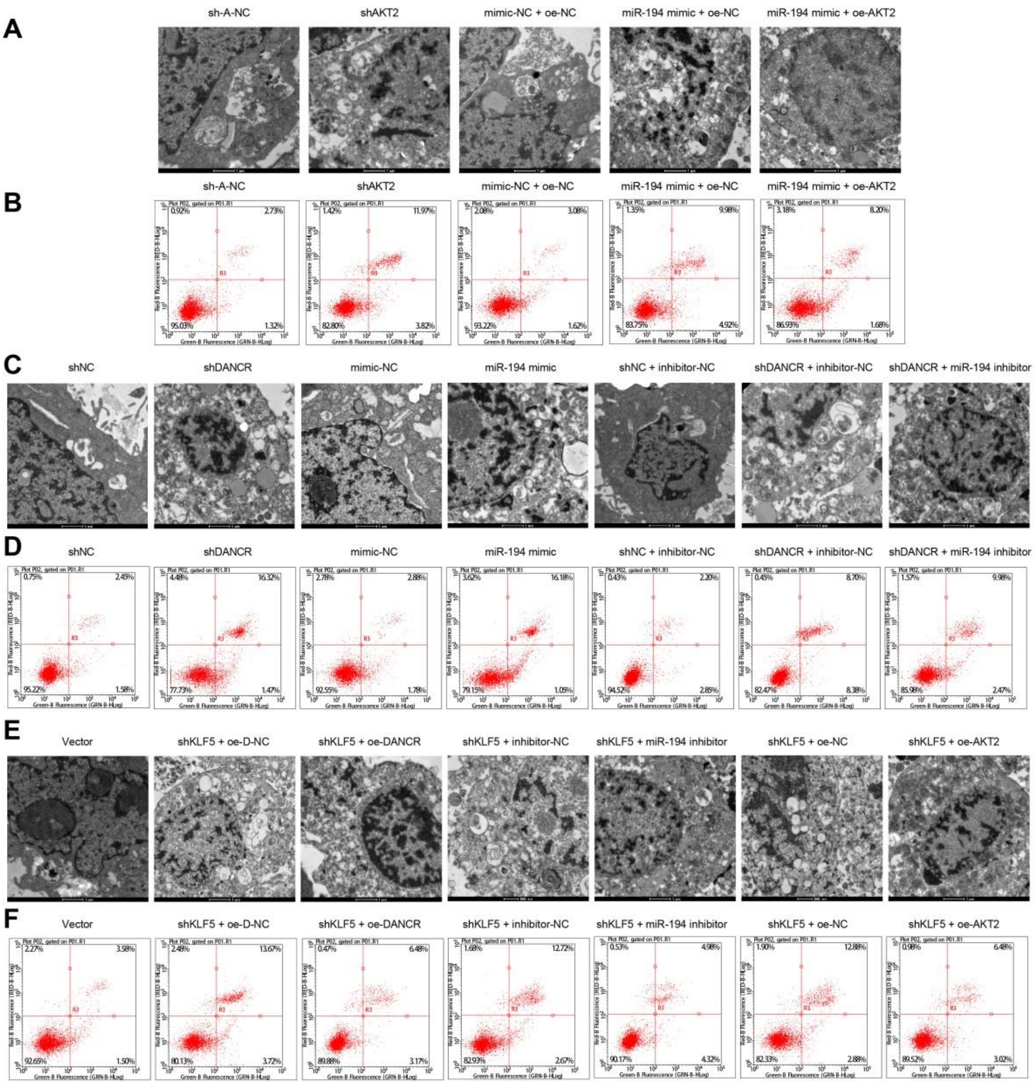

Supplementary Figure 1 KLF5 knockdown promotes GC cell autophagy by regulating the DANCR/miR-194/AKT2 axis. A. Representative images of transmission electron microscope to observe the autophagy of cells in response to altered expression of AKT2 and miR-194; B. Flow cytometric analysis for the apoptosis of cells in response to altered expression of AKT2 and miR-194. C. Representative images of transmission electron microscope to observe the autophagy of cells in response to altered expression of DANCR and miR-194; D. Flow cytometric analysis for the apoptosis of cells in response to altered expression of DANCR and miR-194. E. Representative images of transmission electron microscope to observe the autophagy of cells in response to altered expression of KLF5, DANCR, miR-194 and AKT2; F. Flow cytometric analysis for the apoptosis of cells in response to altered expression of KLF5, DANCR, miR-194 and AKT2.

Supplementary table 1. Correlation between KLF5 expression and clinicopathological features of patients with gastric cancer.

Clinicopathological features	Case (n = 86)	Expression of KLF5		*p*
		Negative (%)	Positive (%)	
Sex				
Male	55	30 (63.6)	25 (36.4)	0.369
Female	31	13 (41.9)	18 (58.1)	
Age (year)				
≤ 60	48	22 (45.8)	26 (54.2)	0.515
> 60	38	21 (55.3)	17 (44.7)	
Tumor size (cm)				
≤ 3	29	23 (79.3)	6 (20.7)	< 0.001
> 3	57	20 (35.1)	37 (64.9)	
TNM				
I ~ II	34	25 (78.1)	9 (21.9)	< 0.001
III ~ IV	52	18 (33.3)	34 (66.7)	
Tumor invasion depth				
T1 + T2	26	21 (84.0)	5 (16.0)	< 0.001
T3 + T4	60	22 (36.1)	38 (63.9)	
Lymph node metastasis				
Presence	50	11 (22.0)	39 (78.0)	< 0.001
Absence	36	32 (88.9)	4 (11.1)	

The results are enumeration data, which were analyzed by Chi-square test. Sample size n = 86. The results were considered highly statistically significant when $p < 0.001$.

Supplementary Table 2. Sequences for shRNAs

shRNA	Sequence
sh-NC	Sense: AATTCTCCGAACGTGTCACGT<br>Antisense: ACGTGACACGTTCGGAGAATT
shDANCR#1	Sense: GATCCTCGGAGGTGGATTCTGTTAGAGTACTGTAACAGAATCCACCTCCGATTTTTTC<br>Antisense: TCGAGAAAAAATCGGAGGTGGATTCTGTTACAGTACTCTAACAGAATCCACCTCCGAG
shDANCR#2	Sense: GATC TAAGCCGGTCATGAGATTATACTCGAGTATAATCTCATGACCGGCTTATTTTTG<br>Antisense: AATTCAAAAATAAGCCGGTCATGAGATTATACTCGAGTATAATCTCATGACCGGCTTA
shDANCR#3	Sense: GATC AGAAGCCTGGCAGGTTAATAACTCGAGTTATTAACCTGCCAGGCTTCTTTTTTG<br>Antisense: AATTCAAAAAAGAAGCCTGGCAGGTTAATAACTCGAGTTATTAACCTGCCAGGCTTCT
shKLF5*1	Sense: GATCCGGATGAATTAACGCGCCATTATTCAAGACGTTACTAATGGCGCGTTAATTCATCCTTTTTTGTCGACA<br>Antisense: AGCTTGTCGACAAAAAAGGATGAATTAACGCGCCATTAGTAACGTCTTGAATAATGGCGCGTTAATTCATCCG
shKLF5*2	Sense: CACCGACGGTCTCTGGGATTTGTAG<br>Antisense: AAACCTACAAATCCCAGAGACCGTC
shKLF5*3	Sense: GGTCCAGACAAGATGTGAAAT<br>Antisense: TTCACATCTTGTCTGGACCAG
shAKT2#1	Sense: GGGCTAAAGTGACCATGAATT<br>Antisense: TTCATGGTCACTTTAGCCCTT
shAKT2#2	Sense: AAGGATGAAGTCGCTCACACA

	Antisense: TGTGTGAGCGACTTCATCCTT
shAKT2#3	Sense: TGACTTCGACTATCTCAAATT<br>Antisense: TTTGAGAUAGTCGAAGTCATT

Supplementary Table 3. qRT-PCR Primer sequences

Gene	Sequence
KLF5	Forward: 5′-CCACCACCCTGCCAGTTAAC-3′<br>Reverse: 5′-TAAACTTTTGTGCAACCAGGGTAA-3′
DANCR	Forward: 5′-AGTTCTGACCACGAGCTTTTC-3′<br>Reverse: 5′-GGTGCTATGAGATTCCGAGTTC-3′
miR-194	Forward: 5′-ACACTCCAGTGGGTGTAAACATCCTCGA-3′<br>Reverse: 5′-TGGTGTCGTGGAGTCG-3′
AKT2	Forward: 5′-GCATGAAACTGGACTGCTCA-3′<br>Reverse: 5′-TGCCTGAAGCTTGTGACATC-3′
U6	Forward: 5′-CTCGCTTCGGCAGCACA-3′<br>Reverse: 5′-TGGTGTCGTGGAGTCG-3′
GAPDH	Forward: 5′-TCCCATCACCATCTTCCA-3′<br>Reverse: 5′-CATCACGCCACAGTTTCC-3′

Figure 1K

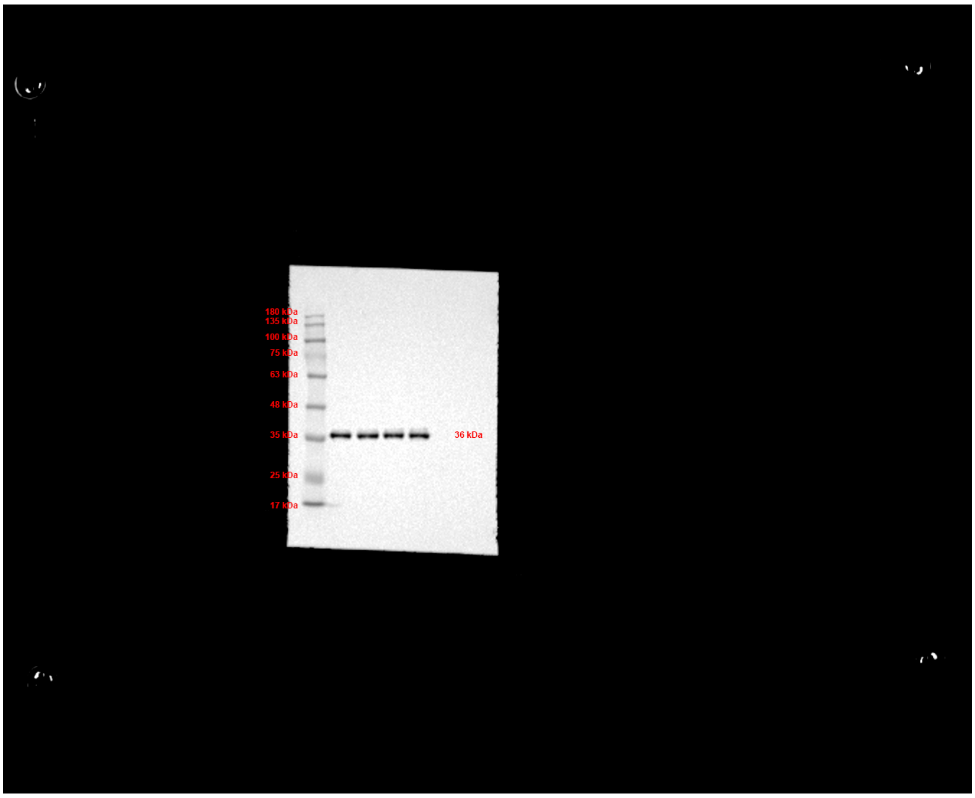

GAPDH

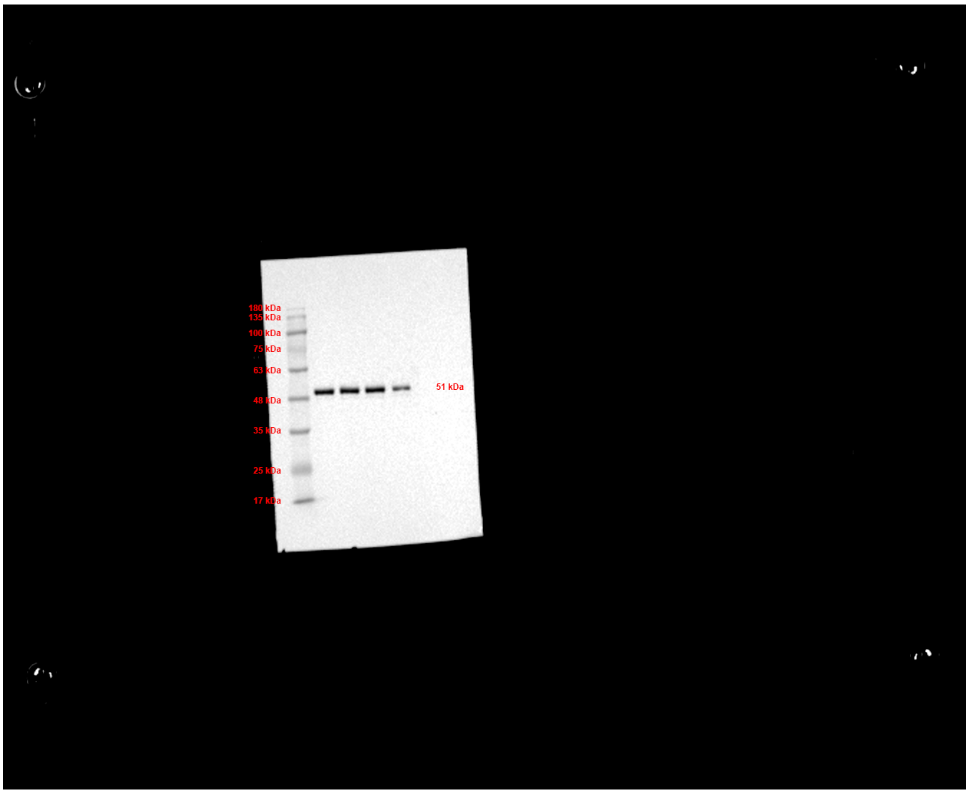

KLF5

Figure 2J

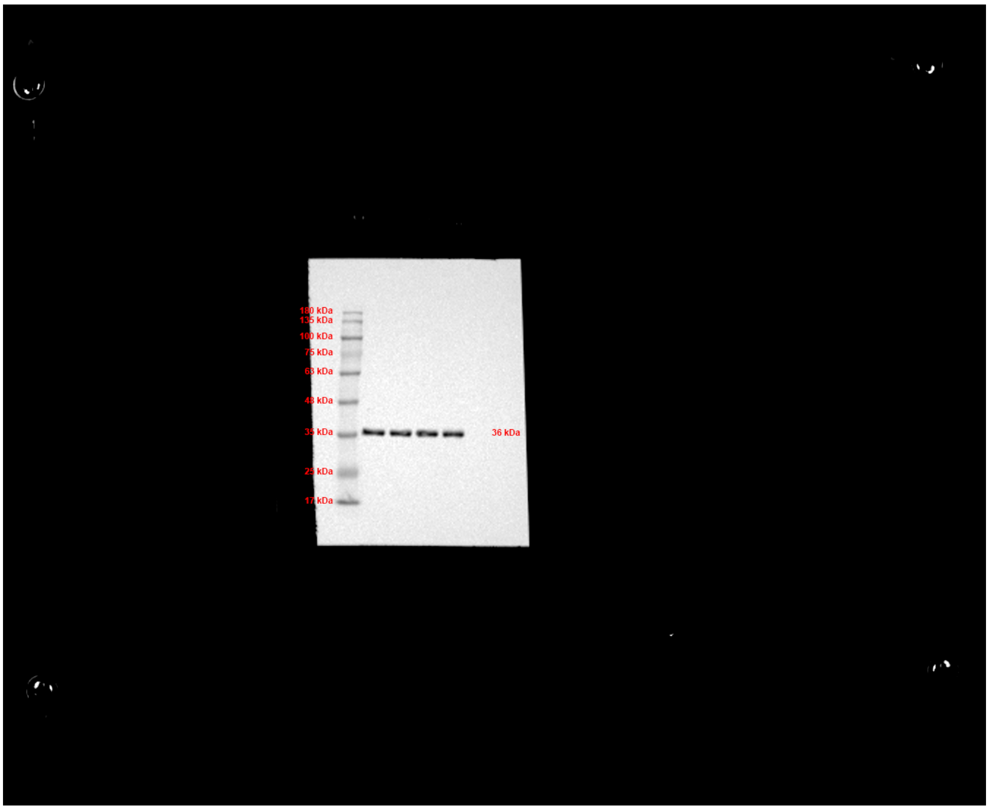

GAPDH

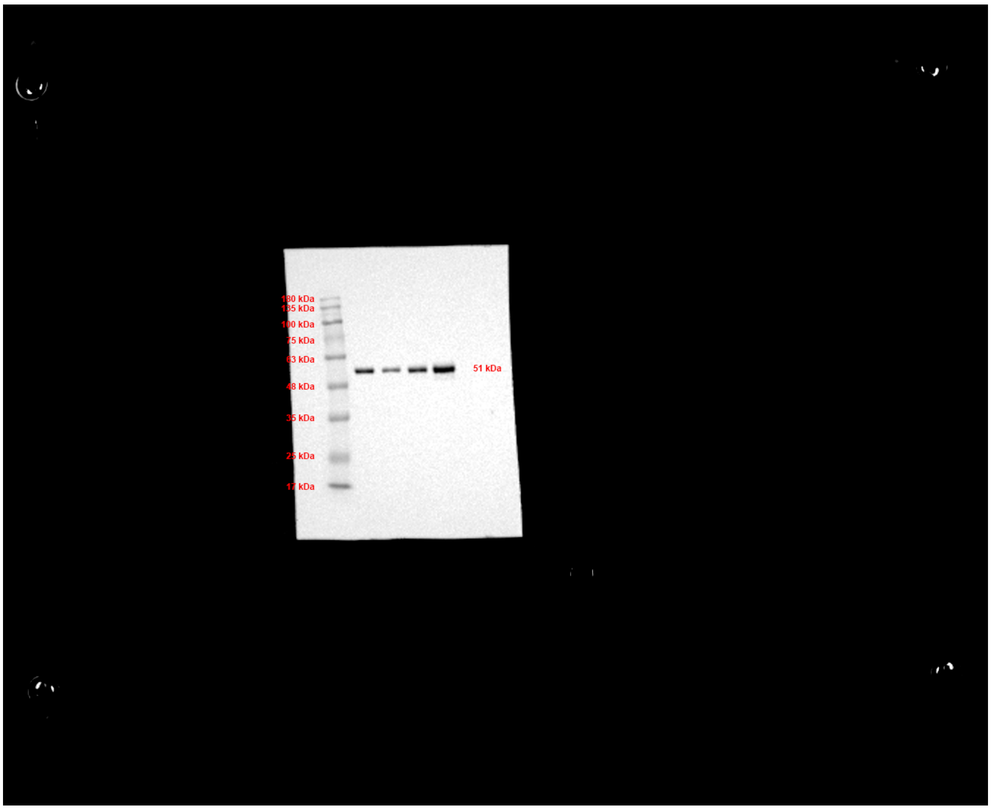

KLF5

Figure 3D

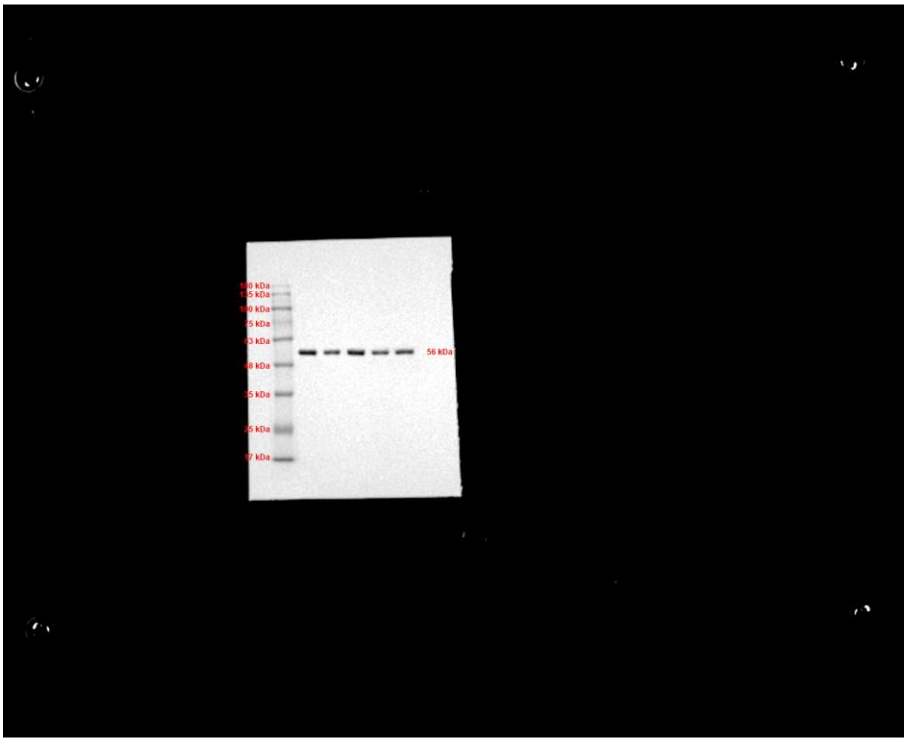

AKT2

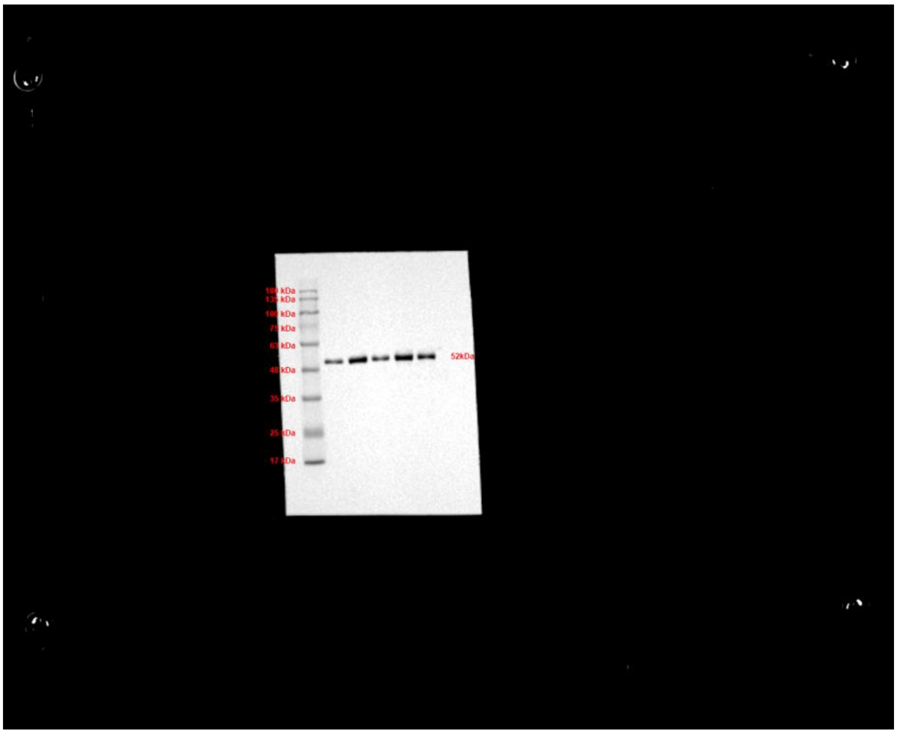

Beclin-1

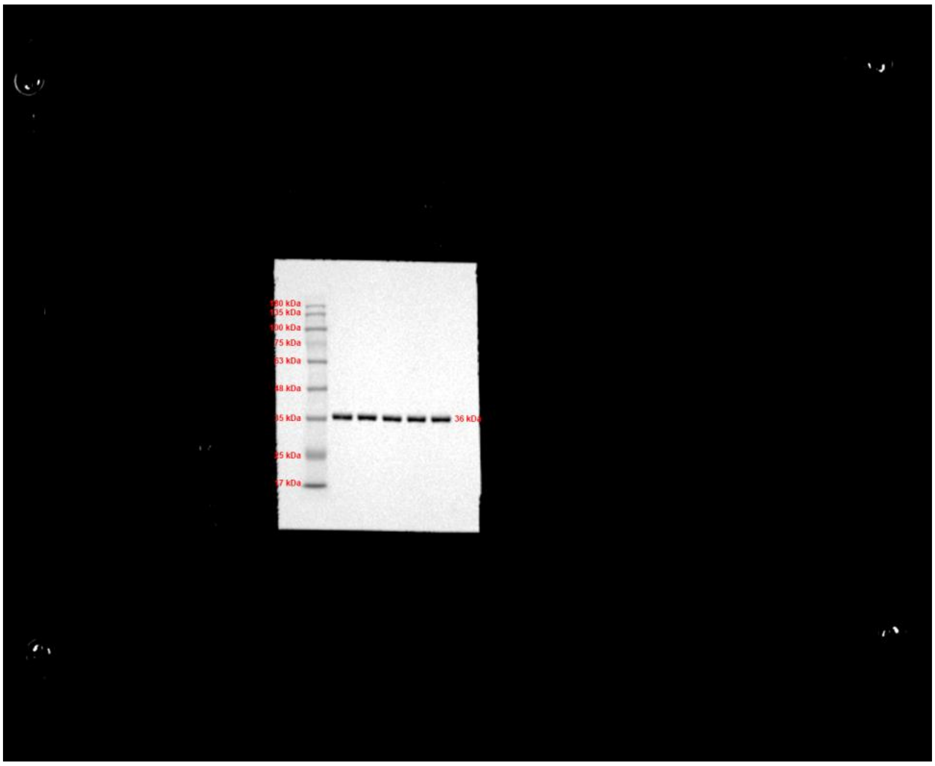

GAPDH

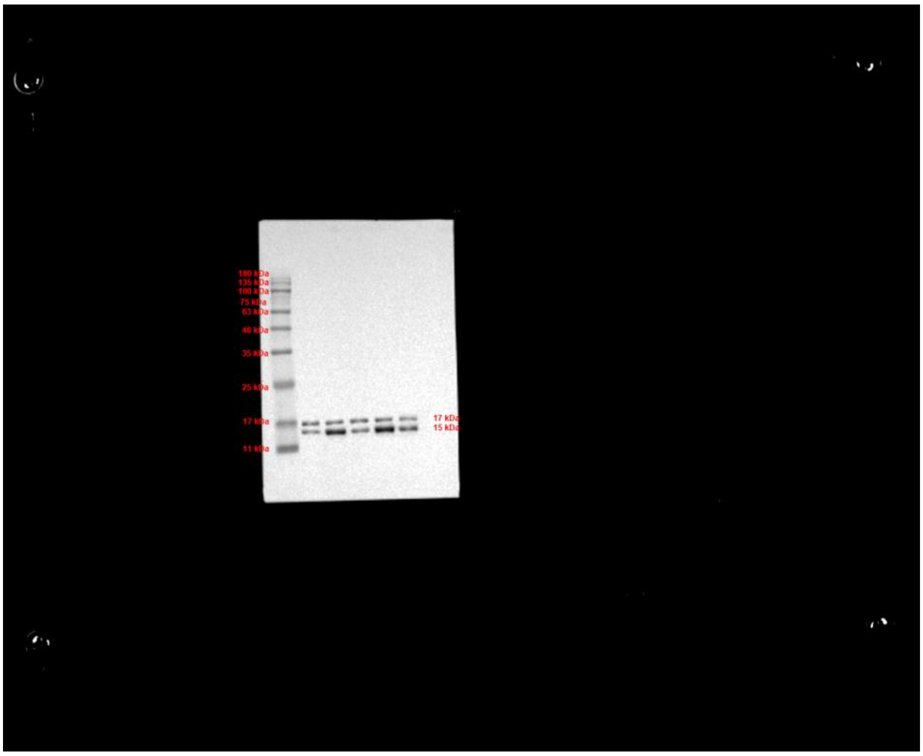

LC3-I and LC3-II

Figure 5D

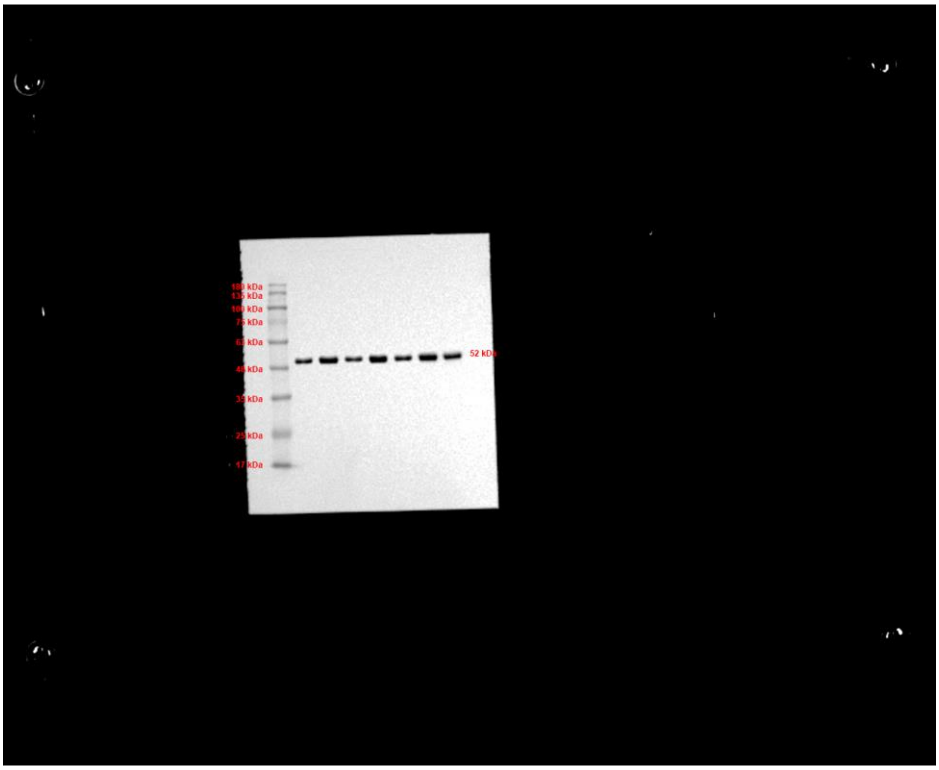

Beclin-1

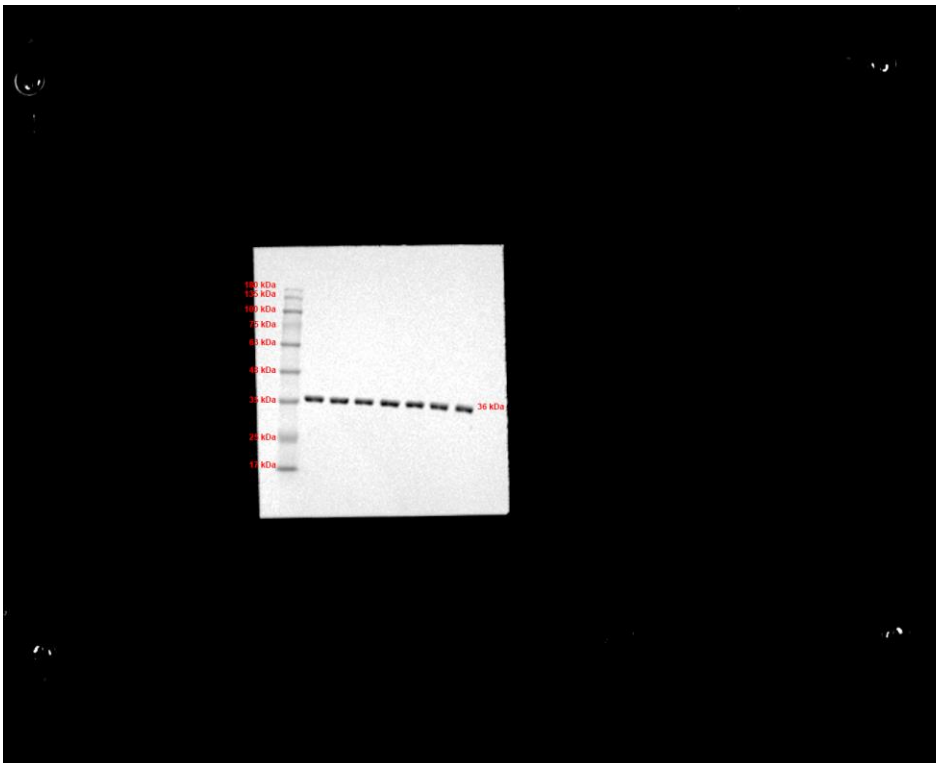

GAPDH

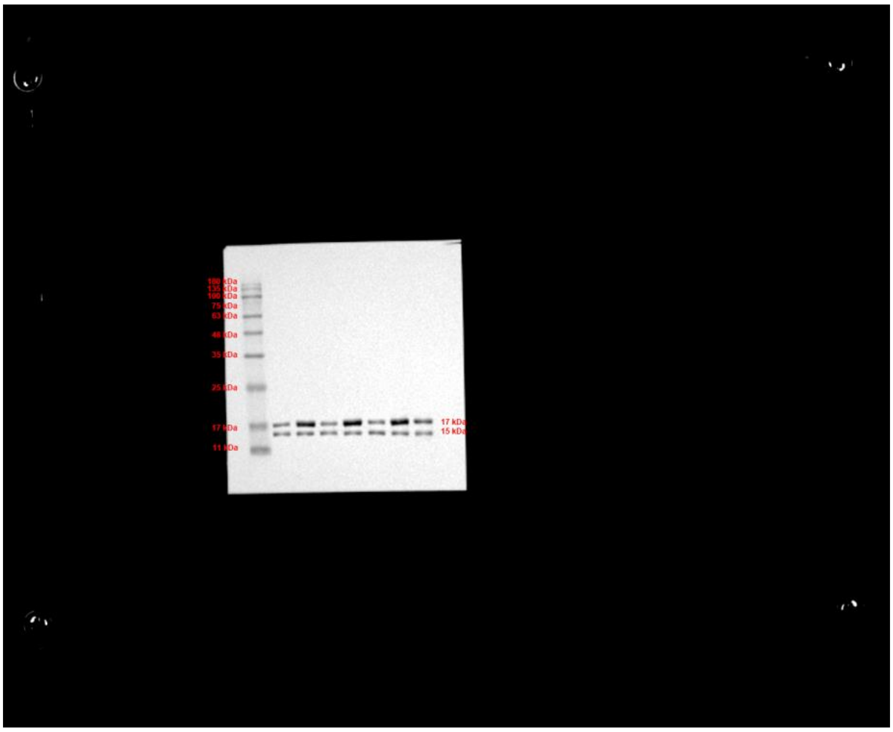

LC3-I and LC3-II

Figure 6D

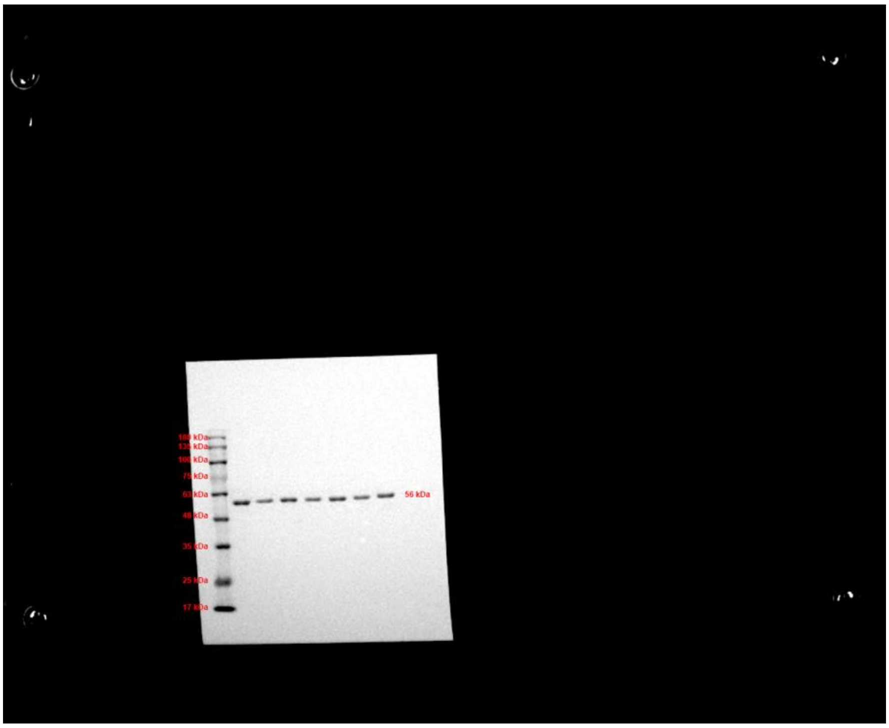

AKT2

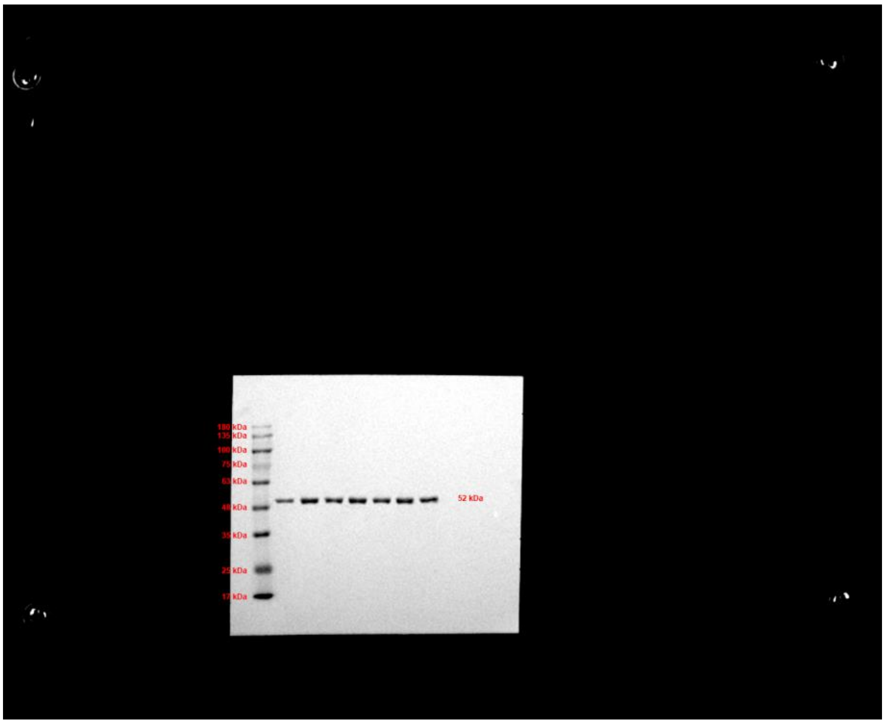

Beclin-1

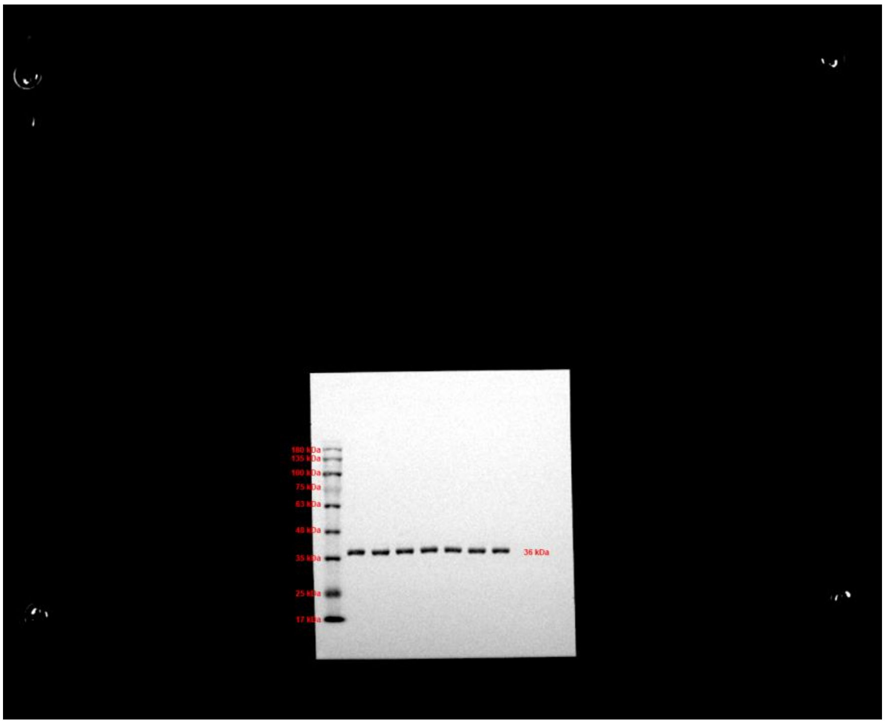

GAPDH

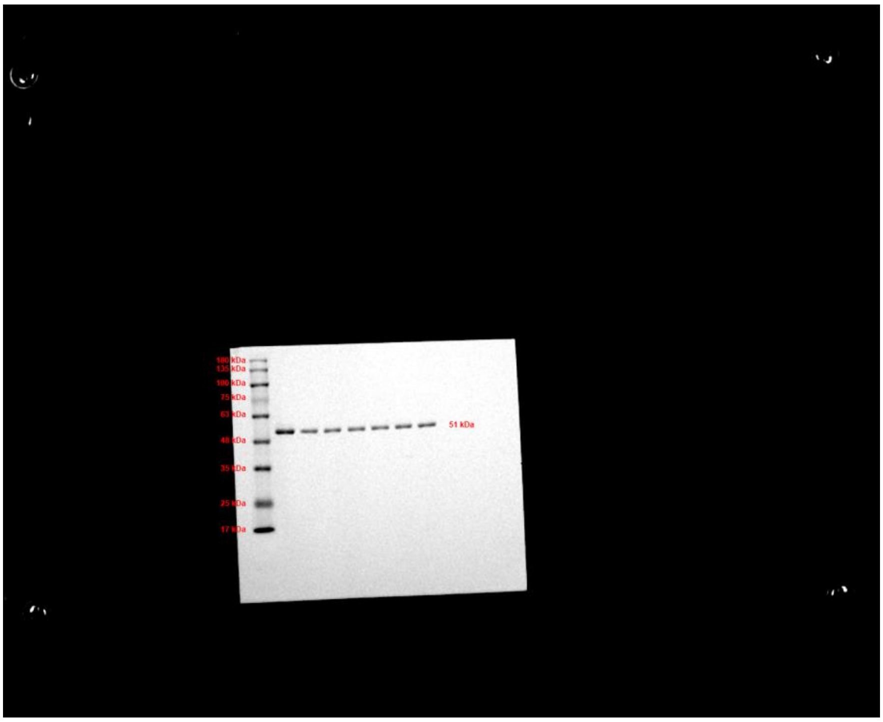

KLF5

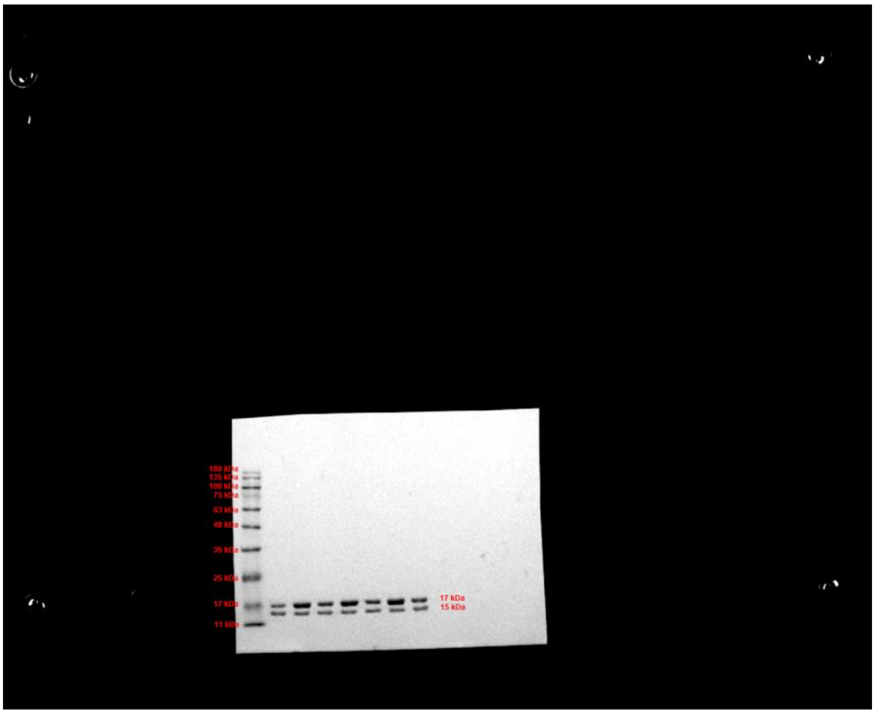

LC3-I and LC3-II
