# Supplementary material for: KLF5 activates lncRNA DANCR and inhibits cancer cell autophagy accelerating gastric cancer progression
Source: NPJ Genom Med. 2021 Sep 21;6:75. doi: 10.1038/s41525-021-00207-7 (PMC8455684; doi:10.1038/s41525-021-00207-7)
Supplement: Supplementary file 2 — Reporting Summary [file 41525_2021_207_MOESM2_ESM.pdf]

nature research

Corresponding author(s): Xiaojun Zhao

Last updated by author(s): Jan 20, 2021

Reporting Summary

Nature Research wishes to improve the reproducibility of the work that we publish. This form provides structure for consistency and transparency in reporting. For further information on Nature Research policies, see our Editorial Policies and the Editorial Policy Checklist.

Statistics

For all statistical analyses, confirm that the following items are present in the figure legend, table legend, main text, or Methods section.

n/a	Confirmed	
☐	☒	The exact sample size (n) for each experimental group/condition, given as a discrete number and unit of measurement
☐	☒	A statement on whether measurements were taken from distinct samples or whether the same sample was measured repeatedly
☐	☒	The statistical test(s) used AND whether they are one- or two-sided<br>*Only common tests should be described solely by name; describe more complex techniques in the Methods section.*
☒	☐	A description of all covariates tested
☒	☐	A description of any assumptions or corrections, such as tests of normality and adjustment for multiple comparisons
☒	☐	A full description of the statistical parameters including central tendency (e.g. means) or other basic estimates (e.g. regression coefficient) AND variation (e.g. standard deviation) or associated estimates of uncertainty (e.g. confidence intervals)
☒	☐	For null hypothesis testing, the test statistic (e.g. F, t, r) with confidence intervals, effect sizes, degrees of freedom and P value noted<br>*Give P values as exact values whenever suitable.*
☒	☐	For Bayesian analysis, information on the choice of priors and Markov chain Monte Carlo settings
☒	☐	For hierarchical and complex designs, identification of the appropriate level for tests and full reporting of outcomes
☒	☐	Estimates of effect sizes (e.g. Cohen's d, Pearson's r), indicating how they were calculated

Our web collection on statistics for biologists contains articles on many of the points above.

Software and code

Policy information about availability of computer code

Data collection	Venn diagram (http://bioinformatics.psb.ugent.be/webtools/Venn/) of databases of microT (http://diana.imis.athena-innovation.gr/DianaTools/index.php?r=microT_CDS/), mirDIP (http://ophid.utoronto.ca/mirDIP/) and TargetScan (http ://www.targetscan.org/vert_72/) to predict target genes of miR-194<br>The binding site between DANCR and miR-194 predicted through the Blast website (https://blast.ncbi.nlm.nih.gov/Blast.cgi?PAGE=MegaBlast&PROGRAM=blastn&BLAST_PROGRAMS=megaBlast&PAGE_TYPE=BlastSearch&BLAST_SPEC=blast2seq&DATABASE=n/a)
Data analysis	Venn diagram (http://bioinformatics.psb.ugent.be/webtools/Venn/) of databases of microT (http://diana.imis.athena-innovation.gr/DianaTools/index.php?r=microT_CDS/), mirDIP (http://ophid.utoronto.ca/mirDIP/) and TargetScan (http ://www.targetscan.org/vert_72/) to predict target genes of miR-194<br>The binding site between DANCR and miR-194 predicted through the Blast website (https://blast.ncbi.nlm.nih.gov/Blast.cgi?PAGE=MegaBlast&PROGRAM=blastn&BLAST_PROGRAMS=megaBlast&PAGE_TYPE=BlastSearch&BLAST_SPEC=blast2seq&DATABASE=n/a)

For manuscripts utilizing custom algorithms or software that are central to the research but not yet described in published literature, software must be made available to editors and reviewers. We strongly encourage code deposition in a community repository (e.g. GitHub). See the Nature Research guidelines for submitting code & software for further information.

Data

Policy information about availability of data

All manuscripts must include a data availability statement. This statement should provide the following information, where applicable:

- Accession codes, unique identifiers, or web links for publicly available datasets
- A list of figures that have associated raw data
- A description of any restrictions on data availability

Provide your data availability statement here.

Field-specific reporting

Please select the one below that is the best fit for your research. If you are not sure, read the appropriate sections before making your selection.

☒ Life sciences ☐ Behavioural & social sciences ☐ Ecological, evolutionary & environmental sciences

For a reference copy of the document with all sections, see nature.com/documents/nr-reporting-summary-flat.pdf

Life sciences study design

All studies must disclose on these points even when the disclosure is negative.

Sample size	The specimens of 86 GC patients diagnosed between January 2010 and January 2014 in the Fifth Affiliated Hospital of Nantong University (Jiangsu Taizhou People's Hospital) were selected as the study subjects.<br>A total of 12 BALB/c nude mice (J004, Better Biotechnology Co., Ltd., Nanjing, China), aged 3-4 weeks old, and weighing 14-18 g, were included for animal experiments.
Data exclusions	The patients were included in this study if ① they had not received radiochemotherapy or other treatment before surgery, and the clinical data were complete; ② the age was 3 to 70 years old; ③ they had no other malignant tumors, no mental abnormalities, or unconscious disorders. The following patients were excluded from this study: ① patients aged < 3 years or > 70 years; ② patients during pregnancy or lactation; ③patients with other severe systemic diseases or malignant tumors.
Replication	Cellular experiment was repeated three times.
Randomization	Nude mice were randomly divided into 2 groups, 6 in each group.
Blinding	The investigators were blinded to group allocation during data collection and/or analysis.

Reporting for specific materials, systems and methods

We require information from authors about some types of materials, experimental systems and methods used in many studies. Here, indicate whether each material, system or method listed is relevant to your study. If you are not sure if a list item applies to your research, read the appropriate section before selecting a response.

Materials & experimental systems

n/a	Involved in the study	
☐	☒	Antibodies
☒	☐	Eukaryotic cell lines
☒	☐	Palaeontology and archaeology
☐	☒	Animals and other organisms
☐	☒	Human research participants
☐	☒	Clinical data
☒	☐	Dual use research of concern

Methods

n/a	Involved in the study	
☒	☐	ChIP-seq
☐	☒	Flow cytometry
☒	☐	MRI-based neuroimaging

Antibodies

Antibodies used	The primary antibody rabbit anti-KLF5 (ab137676, 1: 100) and the secondary antibody IgG (ab205718, 1: 2000) were all purchased from Abcam (Cambridge, UK).<br>The blots were probed with dilute primary antibody rabbit anti-KLF5 (ab137676, 1: 100), AKT2 (ab32505, 1: 3000), LC3B (ab51520, 1: 3000, Abcam, UK), Beclin-1 (ab207612, 1: 2000, Abcam, UK), GAPDH (ab181602, 1: 10000) overnight at 4°C.
Validation	The primary antibody rabbit anti-KLF5 (ab137676, 1: 100) and the secondary antibody IgG (ab205718, 1: 2000) were all purchased from Abcam (Cambridge, UK).

The blots were probed with dilute primary antibody rabbit anti-KLF5 (ab137676, 1: 100), AKT2 (ab32505, 1: 3000), LC3B (ab51520, 1: 3000, Abcam, UK), Beclin-1 (ab207612, 1: 2000, Abcam, UK), GAPDH (ab181602, 1: 10000) overnight at 4°C.

Animals and other organisms

Policy information about studies involving animals; ARRIVE guidelines recommended for reporting animal research

Laboratory animals	12 male BALB/c nude mice (J004, Better Biotechnology Co., Ltd., Nanjing, China), male,aged 3-4 weeks old, and weighing 14-18 g
Wild animals	The study did not involve wild animals
Field-collected samples	The study did not involve samples collected from the field
Ethics oversight	Animal experiments were performed in accordance with Guide for the Care and Use of Laboratory Animals published by the National Institutes of Health, and approved by the Animal Ethics Committee of the Fifth Affiliated Hospital of Nantong University (Jiangsu Taizhou People's Hospital).

Note that full information on the approval of the study protocol must also be provided in the manuscript.

Human research participants

Policy information about studies involving human research participants

Population characteristics	The clinicopathological features (sex, age, tumor size, TNM, tumor invasion depth and lymph node metastasis) of patients involved are presented in Table 1.
Recruitment	The specimens of 86 GC patients diagnosed between January 2010 and January 2014 in the Fifth Affiliated Hospital of Nantong University (Jiangsu Taizhou People's Hospital) were selected as the study subjects, including 55 males and 31 females, with a mean age of 59.85 ± 6.88 years. The patients were included in this study if ① they had not received radiochemotherapy or other treatment before surgery, and the clinical data were complete; ② the age was 3 to 70 years old; ③ they had no other malignant tumors, no mental abnormalities, or unconscious disorders. The following patients were excluded from this study: ① patients aged < 3 years or > 70 years; ② patients during pregnancy or lactation; ③patients with other severe systemic diseases or malignant tumors.
Ethics oversight	This study was approved by the ethics committee of the Fifth Affiliated Hospital of Nantong University (Jiangsu Taizhou People's Hospital), and all patients provided written informed consent.

Note that full information on the approval of the study protocol must also be provided in the manuscript.

Clinical data

Policy information about clinical studies

All manuscripts should comply with the ICMJE guidelines for publication of clinical research and a completed CONSORT checklist must be included with all submissions.

Clinical trial registration	NA
Study protocol	The "patients" section in the "MATERIALS AND METHODS" section
Data collection	The specimens of 86 GC patients diagnosed between January 2010 and January 2014 in the Fifth Affiliated Hospital of Nantong University (Jiangsu Taizhou People's Hospital) were selected as the study subjects. All sections were confirmed pathologically by two experienced pathologists. A portion of the tissue sample was immediately stored in liquid nitrogen. The other part of the tissue sample was fixed with 10% formalin and embedded in paraffin for preservation after routine dehydration. These patients were followed up for 60 months.
Outcomes	Primary outcome measure is we verified by qRT-PCR that the expression of KLF5 and DANCR in GC tissues was higher than that in adjacent tissues；<br>secondary outcome measures is immunohistochemical analysis found that the positive expression of KLF5 was brownish yellow, and the expression of KLF5 in GC tissue was higher than that in adjacent tissues；

Flow Cytometry

Plots

Confirm that:

☒ The axis labels state the marker and fluorochrome used (e.g. CD4-FITC).

☒ The axis scales are clearly visible. Include numbers along axes only for bottom left plot of group (a 'group' is an analysis of identical markers).

☒ All plots are contour plots with outliers or pseudocolor plots.

☒ A numerical value for number of cells or percentage (with statistics) is provided.

Methodology

Sample preparation	Three GC cell lines (SGC7901, MGC-803, NCI-N87) and one normal gastric epithelial cell (GES-1) were purchased from the Cell Bank of the Chinese Academy of Sciences (Shanghai, China).
Instrument	The flow cytometer (BIO-RAD, Hercules, CA, USA) was employed to evaluate cell apoptosis.
Software	Bio-Rad ZE5， BIO-RAD
Cell population abundance	The abundance of cell population was 6000 and the purity of cell was 0.6.
Gating strategy	In the first gate, FSC / SSC was used to eliminate cell debris and noise interference. In the second gate, negative control and positive control were used as reference to set the gate position.

☐ Tick this box to confirm that a figure exemplifying the gating strategy is provided in the Supplementary Information.
