# Supplementary material for: KLF5 activates lncRNA DANCR and inhibits cancer cell autophagy accelerating gastric cancer progression
Source: NPJ Genom Med. 2021 Sep 21;6:75. doi: 10.1038/s41525-021-00207-7 (PMC8455684; doi:10.1038/s41525-021-00207-7)
Supplement: Supplementary file 3 — Supplementary Table 1 [file 41525_2021_207_MOESM3_ESM.docx]

**Supplementary Table 1 |** qRT-PCR Primer sequences

| Gene | Primer sequence |
| --- | --- |
| KLF5 | Forward: 5′-CCACCACCCTGCCAGTTAAC-3′ |
|  | Reverse: 5′-TAAACTTTTGTGCAACCAGGGTAA-3′ |
| DANCR | Forward: 5′-AGTTCTGACCACGAGCTTTTC-3′ |
|  | Reverse: 5′-GGTGCTATGAGATTCCGAGTTC-3′ |
| miR-194 | Forward: 5′-ACACTCCAGTGGGTGTAAACATCCTCGA-3′ |
|  | Reverse: 5′-TGGTGTCGTGGAGTCG-3′ |
| AKT2 | Forward: 5′-GCATGAAACTGGACTGCTCA-3′ |
|  | Reverse: 5′-TGCCTGAAGCTTGTGACATC-3′ |
| U6 | Forward: 5′-CTCGCTTCGGCAGCACA-3′ |
|  | Reverse: 5′-TGGTGTCGTGGAGTCG-3′ |
| GAPDH | Forward: 5′-TCCCATCACCATCTTCCA-3′ |
|  | Reverse: 5′-CATCACGCCACAGTTTTCC-3′ |
